# RAD51 and mitotic function of mus81 are essential for recovery from low-dose of camptothecin in the absence of the WRN exonuclease

**DOI:** 10.1093/nar/gkz431

**Published:** 2019-05-22

**Authors:** Francesca Antonella Aiello, Anita Palma, Eva Malacaria, Li Zheng, Judith L Campbell, Binghui Shen, Annapaola Franchitto, Pietro Pichierri

**Affiliations:** 1 Mechanisms, Biomarkers and Models Unit, Department of Environment and Health, Istituto Superiore di Sanità, Roma, Italy; 2 Department of Cancer Genetics and Epigenetics, Beckman Research Institute, City of Hope, 1500 East Duarte Road, Duarte, CA 91010–3000, USA; 3 Braun Laboratories, California Institute of Technology, Pasadena, CA 91125, USA; 4 Istituto Nazionale Biostrutture e Biosistemi, Roma, Italy

## Abstract

Stabilization of stalled replication forks prevents excessive fork reversal or degradation, which can undermine genome integrity. The WRN protein is unique among the other human RecQ family members to possess exonuclease activity. However, the biological role of the WRN exonuclease is poorly defined. Recently, the WRN exonuclease has been linked to protection of stalled forks from degradation. Alternative processing of perturbed forks has been associated to chemoresistance of BRCA-deficient cancer cells. Thus, we used WRN exonuclease-deficiency as a model to investigate the fate of perturbed forks undergoing degradation, but in a BRCA wild-type condition. We find that, upon treatment with clinically-relevant nanomolar doses of the Topoisomerase I inhibitor camptothecin, loss of WRN exonuclease stimulates fork inactivation and accumulation of parental gaps, which engages RAD51. Such mechanism affects reinforcement of CHK1 phosphorylation and causes persistence of RAD51 during recovery from treatment. Notably, in WRN exonuclease-deficient cells, persistence of RAD51 correlates with elevated mitotic phosphorylation of MUS81 at Ser87, which is essential to prevent excessive mitotic abnormalities. Altogether, these findings indicate that aberrant fork degradation, in the presence of a wild-type RAD51 axis, stimulates RAD51-mediated post-replicative repair and engagement of the MUS81 complex to limit genome instability and cell death.

## INTRODUCTION

The response to perturbed replication is crucial for the maintenance of genome integrity (1–5). In humans, proper handling of perturbed replication forks is also linked to cancer avoidance and many proteins involved in this process act as onco-suppressors (3–6). The importance of correctly dealing with perturbed replication forks is also demonstrated by the existence of several human genetic diseases caused by mutations in factors that sense, process and recover replication forks (7).

The Werner's syndrome protein (WRN) is one of these key factors, and is mutated in the genetic disease Werner's syndrome (WS), which is characterized by cancer predisposition and premature aging (8,9). From an enzymatic point of view, WRN is both a DNA helicase and exonuclease; however, while its helicase activity has been linked to processing of reversed or collapsed replication forks (2,9), little is known about the biological relevance of the exonuclease activity. Loss of WRN confers sensitivity to several DNA-damaging agents inducing replication stress, including Topoisomerase inhibitors (8,10,11). We recently reported that the exonuclease activity of WRN is involved in protecting replication forks perturbed by treatment with the Topoisomerase I poison Camptothecin (CPT) in the nanomolar range of concentration (12). Exposure to low doses of CPT, as opposed to high doses, does not induce DSBs but stimulates greatly formation of reversed forks (13,14). Reversed replication forks are versatile yet vulnerable structures and several proteins participate in their stabilisation (15–17). Two proteins, BRCA2 and RAD51, are the most crucial for the stabilisation of reversed forks (15,17,18). Thus, cells depleted of each of these two proteins have been used as a prototypical model to assess the consequences of inaccurate handling of reversed forks. However, BRCA2 and RAD51 may also participate in DNA repair, which may be used to fix damage generated by fork instability (18–20). Cells expressing the exonuclease-dead WRN retain ability to restart replication and are not overtly sensitive to low doses of CPT, suggesting that alternative mechanisms can be activated as a back-up. Since nanomolar doses of CPT are clinically-relevant in cancer therapy, cells expressing a catalytically-inactive WRN exonuclease can be used as a model to investigate the fate of CPT-perturbed replication forks undergoing pathological degradation but in a BRCA2-RAD51 wild-type background.

Here, we report that loss of WRN exonuclease channels cells through a pathological RAD51-dependent mechanism that makes perturbed replication forks resistant to breakage upon prolonged exposure to nanomolar dose of CPT. Furthermore, our data suggest that enhanced accumulation of ssDNA and recruitment of RAD51 interfere with correct activation of CHK1, which provides a positive feedback to the formation of nascent ssDNA. Pathological engagement of RAD51 makes WRN exonuclease-deficient cells dependent on the mitotic function of the MUS81 complex to mitigate mitotic abnormalities deriving from accumulation of RAD51-dependent intermediates.

## MATERIALS AND METHODS

### Cell lines and culture conditions

The SV40-transformed WRN-deficient fibroblast cell line (AG11395) was obtained from Coriell Cell Repositories (Camden, NJ, USA). To produce stable cell lines, AG11395 (WS) fibroblasts were transduced with retroviruses expressing the full-length cDNA encoding wild-type WRN (WS^WT^), exonuclease-dead (WS^E84A^) or helicase-dead (WS^K577M^) (21). All the cell lines were maintained in Dulbecco's modified Eagle's medium (DMEM; Life Technologies) supplemented with 10% FBS (Boehringer Mannheim) and incubated at 37°C in a humidified 5% CO_2_ atmosphere.

### Plasmids transfection

Plasmid expressing the wild-type (Flag-CHK1^WT^) or the phospho - mimic (Flag-CHK1^S317/345D^) mutant form of CHK1, a kind gift from Professor K.K. Khanna (Queensland Institute of Medical Research, Australia) was generated as described (22). To express the plasmids, cells were transfected using the Neon™ Transfection System Kit (Invitrogen), according to the manufacturer's instructions.

### Immunofluorescence assays

Cells were grown on 35-mm coverslips and harvested at the indicated times after treatments. For RAD51 and pS345CHK1 IF, after further washing with PBS, cells were pre-extracted with 0,5% TritonX-100 and fixed with 3% PFA/2% sucrose at RT for 10 min. After blocking in 3% BSA for 15 min, staining was performed with rabbit monoclonal anti-RAD51 or anti- pS345CHK1 diluted in a 1% BSA/0.1% saponin in PBS solution, for 1 h at 37°C in a humidified chamber. For 53BP1, pS87MUS81, α-tubulin, Cyclin A, pS10H3 and pThr1989ATR staining, cells were fixed with 4% PFA at RT for 10 min. Cells were subsequently permeabilized with 0.4% Triton-X100 and blocked with 3% BSA. Staining with primary antibodies diluted in a 1% BSA/0.1% saponin in PBS solution was carried out for 1 h at 37° in a humidified chamber. After extensive washing with PBS, specie-specific fluorophore-conjugated antibody (Invitrogen) was applied for 1h at RT followed by counterstaining with 0.5 mg/ml DAPI. Secondary antibody was used at 1:200 dilution. Images were acquired as grayscale files using Metaview software (MDS Analytical Technologies) and processed using Adobe Photoshop CS3 (Adobe). For each time point, at least 200 nuclei were examined, and foci were scored at 40×. Only nuclei with >5 foci were considered as positive and were quantified using ImageJ.

### Antibodies

The primary antibodies used were: anti-pS345CHK1 (1:100, Cell Signaling Technologies), anti-pThr1989ATR (1:100, GeneTex) anti-pS10H3 (1:1000, Santa Cruz Biotechnologies), anti-Cyclin A (IF: 1:100, Santa Cruz Biotechnologies), anti-53BP1 (1:300, Millipore), anti-BrdU (1:50, Becton Dickinson; anti-IdU detection), anti-pS87MUS81 (WB 1:1000, IF 1:200, Abgent), anti-RAD51 (WB 1:1000, IF 1:100 Bioss Antibodies), anti-α-Tubulin (1:50, Sigma-Aldrich), anti-Flag (1:1000, Sigma-Aldrich) and anti-Lamin B1 (1:10000, Abcam). HRP-conjugated matched secondary antibodies were from Jackson Immunoresearch and were used at 1:20 000.

### Chromatin fractionation and western blot analysis

Chromatin fractionation experiments were performed as previously described (23). Western blotting was performed using standard methods. Blots were incubated with primary antibodies against: rabbit anti-pCHK1(S345) (Cell Signalling Technology), mouse anti-CHK1 (Santa Cruz Biotechnology), rabbit anti-RAD51 (Bioss Antibodies), mouse anti-RPA32 (Calbiochem), rabbit anti-RPA70 (GeneTex), mouse anti-GAPDH (Millipore) and rabbit anti-Lamin B1 (Abcam). After incubations with horseradish peroxidase-linked secondary antibodies (1:20 000, Jackson Immunosciences), the blots were developed using the chemiluminescence detection kit WesternBright ECL HRP substrate (Advansta) according to the manufacturer's instructions. Quantification was performed on scanned images of blots using the Image Lab software, and the values shown on the graphs represent normalization of the protein content evaluated through LaminB1 or GAPDH immunoblotting.

### Clonogenic survival

Cells were plated onto 35 mm dishes, after 24 h they were treated with different doses of CPT. After 18, cells were washed, trypsinized and seeded in 60mm dishes. After 14–21 days, plates were stained with crystal violet and colonies counted.

### Detection of nascent single-stranded DNA

To detect nascent single-stranded DNA (ssDNA), cells were plated onto 22 × 22 coverslips in 35 mm dishes. After 24 h, the cells were labeled for 15 min before the treatment with 150 μM IdU (Sigma-Aldrich), cells were then treated with CPT 50 nM or 5 μM for different time points. Next, cells were washed with PBS, permeabilized with 0.5% Triton X-100 for 10 min at 4°C and fixed wit 2% sucrose, 3% PFA. For ssDNA detection, cells were incubated with primary mouse anti-BrdU antibody (Becton Dickinson) for 1 h at 37°C in 1% BSA/0.1% saponin, followed by Alexa Fluor488-conjugated goat-anti-Mouse (Invitrogen), and counterstained with 0.5 μg/ml DAPI. Slides were analyzed with Eclipse 80i Nikon Fluorescence Microscope, equipped with a VideoConfocal (ViCo) system. For each time point, at least 100 nuclei were scored at 40×. Parallel samples either incubated with the appropriate normal serum or only with the secondary antibody confirmed that the observed fluorescence pattern was not attributable to artefacts. Fluorescence intensity for each sample was then analyzed using ImageJ software.

### Statistical analysis

All the data are presented as means of at least two independent experiments. Statistical comparisons of WS^WT^ or WRN-mutant cells to their relevant control were analyzed by ANOVA or Mann–Whitney test. *P* < 0.05 was considered as significant.

## RESULTS

### Loss of WRN exonuclease activity leads to a persisting and unusual formation of nascent ssDNA which compromises formation of DSBs in response to a low-dose of camptothecin

Treatment with nanomolar concentrations of CPT does not induce DSBs immediately but stimulates fork reversal (13). Under such conditions, loss of WRN exonuclease activity results in the rapid (<2 h) degradation of both nascent strands by the MRE11-EXO1 nucleases (12). In this regard, WRN exonuclease deficiency is a useful model to determine what happens to nuclease-targeted forks after prolonged treatment with a low dose of CPT in a genetic background retaining BRCA2-RAD51 function.

**Figure 1.**
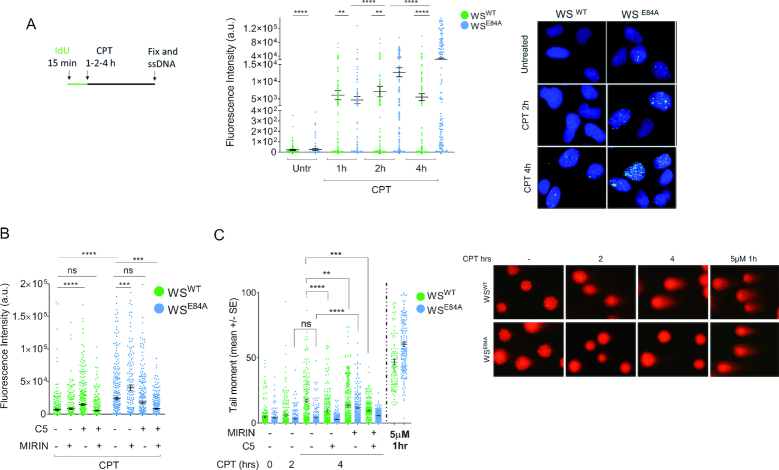
Loss of WRN exonuclease activity leads to formation of nascent ssDNA which compromises formation of DSBs in response to a low-dose of camptothecin. (**A**) Evaluation of ssDNA by anti-IdU immunofluorescence under non-denaturing condition. Nascent DNA was pre-labeled for 15 min with IdU before treatment and labeling remained during treatment with CPT. Dot plots show the mean intensity of ssDNA staining for single nuclei from cells expressing the wild-type (WS^WT^) or the exo-dead form of WRN (WS^E84A^). Cells were either left untreated or challenged with 50 nM CPT for increasing periods, as indicated. The intensity of the anti-IdU immunofluorescence was measured in at least 200 nuclei from three independent experiments. Values are represented as means ±SE. Representative images of ssDNA labeling are shown. (**B**) Evaluation of nascent ssDNA in cells treated with nuclease inhibitors. Cells were treated with Mirin, C5 or both 30 min before IdU labeling and 45 min before CPT treatment for 4 h, and then subjected to the ssDNA assay. The graph shows the mean intensity of IdU fluorescence measured from two independent experiments (*n* = 200), data are presented as mean ± SE. Statistical analysis in A and B was performed by the Mann–Whitney test (**P < 0.01; ***P < 0.001; ****P < 0.0001). (**C**) Analysis of DSB accumulation by the neutral Comet assay. Cells were treated or not with CPT 50 nM for the indicated time, or with 5μM CPT (high-dose) for 1 h, and then subjected to the neutral Comet assay. Where indicated, cells were pre-treated with Mirin, C5 or both. In the graph, data are presented as mean tail moment ± SE from two independent experiments (ns = not significant; **P < 0.01; ***P < 0.001; ****P < 0.0001; ANOVA test). Representative images from the neutral Comet assay are shown.

We first examined the accumulation of nascent ssDNA as a sign of fork degradation by the native IdU assay. In order to track accumulation of fork processing events with time, we used continuous labeling of nascent DNA and analyzed formation of ssDNA at forks beyond the 2 h of treatment already explored on CPT (Figure 1A; (12)). As expected, WS cells expressing the exo-dead WRN protein (WS^E84A^) showed less nascent ssDNA then the corrected wild-type counterpart (WS^WT^) at 1 h of treatment (Figure 1A). Surprisingly, while the amount of nascent ssDNA increased only slightly in cells expressing the wild-type WRN, it accumulated greatly over time in WRN exonuclease-deficient cells and largely exceeded the wild-type level at 4 h (Figure 1A). Of note, the excessive nascent ssDNA formation detected at 4 h of CPT was recapitulated also in doxycycline-inducible shWRN cells complemented with the RNAi-resistant WRN^E84A^ or the WRN^K577M-E84A^ mutant, which contains mutations disabling both the helicase and the exonuclease activities (Supplementary Figure S1A, B). These results indicate that the observed phenotype is cell line-independent and does not derive from uncoordinated action of helicase and exonuclease activities of WRN. The enhanced accumulation of nascent ssDNA was also independent on any difference in the expression of exo-dead WRN compared to the wild-type (12). Indeed, overexpression of the exo-dead WRN induced aberrant accumulation of ssDNA also in the inducible shWRN cells cultured in the absence of doxycycline (Supplementary Figure S2A, B). Finally, the aberrant accumulation of nascent ssDNA in WS^E84A^ cells was specific of the response to a low-dose of CPT, as treatment with HU or a high-dose of CPT failed to recapitulate the phenotype (Supplementary Figure S3).

Interestingly, when the formation of nascent ssDNA was evaluated using the same labeling scheme used previously in WS^E84A^ cells (Supplementary Figure S4A; (12)), a biphasic curve was clearly apparent (Supplementary Figure S4B). The biphasic curve of ssDNA accumulation after CPT showed an initial increase followed by a drop, consistently with previous data (12), and a subsequent significant increase at the late time-point (Supplementary Figure S4B). This suggests that extensive degradation is maintained over the entire duration of treatment. Furthermore, time-course analysis of the nascent ssDNA formation by the native IdU assay confirmed that Mirin treatment decreases nascent ssDNA exposure early after CPT exposure, while increases ssDNA formation at 4h in WS^E84A^ cells (Supplementary Figure S4C, D), suggesting that late formation of nascent ssDNA occurs through a distinct mechanism.

Since nascent strand degradation is MRE11-dependent in the absence of the WRN exonuclease while it is DNA2-dependent in wild-type cells (12,24), we next examined if chemical inhibition of the two nucleases might reduce the idiosyncratic accumulation of ssDNA detected at 4h of treatment in WS^E84A^ cells. Mirin treatment barely reduced ssDNA detected at 4 h of treatment in wild-type cells (Figure 1B). Surprisingly, Mirin did not decrease the formation of ssDNA in WRN exonuclease-deficient cells but rather increased it even further (Figure 1B). Inhibition of DNA2 by the small-molecule inhibitor C5 (25) increased formation of nascent ssDNA in wild-type but not in WRN exonuclease-deficient cells (Figure 1B). In contrast, concomitant inhibition of DNA2 and MRE11 was ineffective in modulating nascent ssDNA formation in wild-type cells while decreased its level in WS^E84A^ cells (Figure 1B). Of note, ssDNA derived from end-resection of DSBs induced by a micromolar dose of CPT was efficiently reduced by DNA2 inhibitor C5 (Supplementary Figure S5A, B), providing a functional proof of the inactivation of the nuclease activity in our cell model. Depletion of MRE11 or DNA2 by specific siRNA (Supplementary Figure S6A, C) confirmed that, in WS^E84A^ cells, nascent ssDNA was increased when MRE11, but not DNA2, is defective (Supplementary Figure S6B, D). Of note, however, depletion of EXO1 substantially reduced the accumulation of nascent ssDNA at 4 h of CPT, almost restoring the wild-type levels (Supplementary Figure S7A, B). These results indicate that different sets of nucleases are involved in the degradation of nascent ssDNA in WRN exonuclease-deficient cells when treatment is prolonged.

DNA breakage can occur even in response to low-doses of CPT if treatment is sufficiently prolonged (13,14). Thus, to further investigate the origin of the late nascent ssDNA in the WRN exonuclease-deficient cells and the role of the different nucleases, we analyzed the presence of DSBs after treatment with nanomolar CPT by neutral Comet assay. As shown in Figure 1C, treatment with 50nM CPT for 4 h induced some DSBs in wild-type cells, although they are very low compared with those generated by the 5μM reference dose and were not dependent on MUS81 (Supplementary Figure S5C). In contrast, no DSBs were detected in WRN exonuclease-deficient cells after treatment with a low-dose of CPT, even if they were readily seen in response to a high-dose of the drug (Figure 1C).

Interestingly, pre-treatment with Mirin, which enhances ssDNA in WS^E84A^ cells (Figure 1B), resulted in DSBs (Figure 1C). In contrast, Mirin or the DNA2 inhibitor C5 decreased formation of DSBs in wild-type cells, alone or as a combination (Figure 1C). Interestingly, inhibition of both MRE11 and DNA2, which decreases ssDNA formation in WS^E84A^ cells (Figure 1B), significantly reduced DSBs generated by Mirin also in the mutant cells (Figure 1C).

Collectively, our results indicate that loss of the WRN exonuclease leads to accumulation of nascent ssDNA when treatment with nanomolar CPT is prolonged beyond 2 h and that it makes cells refractory to formation of DSBs by CPT. Our data also suggest that the late accumulation of nascent ssDNA in WS^E84A^ cells is related to the activity of multiple nucleases.

### Loss of WRN exonuclease stimulates engagement of RAD51 after CPT-induced fork perturbation

In WRN exonuclease-deficient cells, the late reappearance and accumulation of nascent ssDNA together with loss of DSBs might correlate with engagement of an alternative or aberrant fork processing mode over time after CPT. Since extended regions of ssDNA are a substrate of RAD51 during recombination, we investigated whether loss of WRN exonuclease could affect recruitment of RAD51.

**Figure 2.**
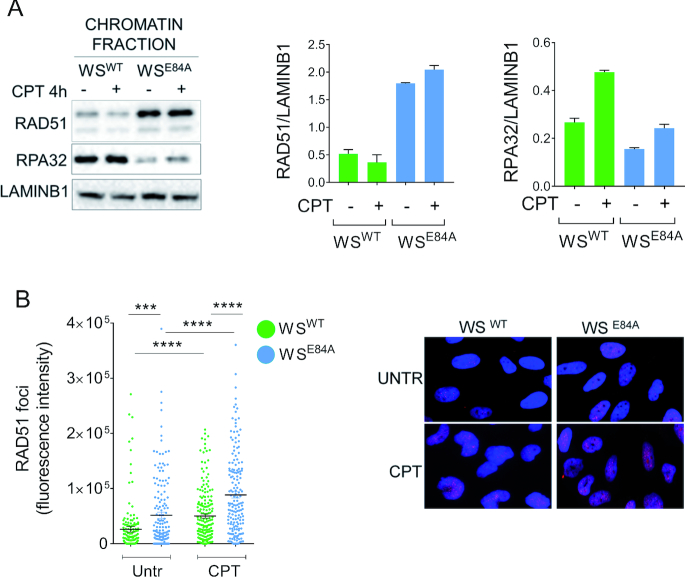
Loss of WRN exonuclease stimulates engagement of RAD51 after CPT. (**A**) WB analysis of chromatin association of RAD51 and RPA32 in wild-type (WS^WT^) and in cells expressing the exo-dead mutant form of WRN (WS^E84A^). Cells were treated or not with CPT for 4 h. LaminB1 was used as loading control. The blot is representative of three replicates. The graphs show the quantification of the amount of RAD51 or RPA32 normalized against LaminB1 (mean ± SE). (**B**) Quantitative immunofluorescence analysis of RAD51 foci in WS^WT^ and WS^E84A^ cells. Cells were treated with CPT 50 nM for 4 h, triton-extracted and subjected to RAD51 immunostaining. Graph shows the intensity of RAD51 immunostaining for each cell with scorable foci (*n* > 3). Values are presented as means ± SE (***P < 0.001; ****P < 0.0001; Mann–Whitney test). Representative images are shown.

We first evaluated recruitment of RAD51 in chromatin using biochemical fractionation and western blotting. In wild-type cells, RAD51 barely increased its chromatin association after CPT treatment (Figure 2A). Expression of the exo-dead WRN, however, greatly heightened the amount of RAD51 in chromatin already in untreated cells and treatment with CPT led to a minimal increase over untreated (∼20%; Figure 2A). As a control, we also measured the level of RPA32 in chromatin. RPA32 is a subunit of the RPA heterotrimer that binds to ssDNA competing with RAD51. Chromatin-bound RPA32 increased in wild-type cells after CPT (Figure 2A). In contrast, the amount of RPA32 in chromatin was low in the absence of the WRN exonuclease and did not show any increase after treatment (Figure 2A). Immunofluorescence analysis of RPA32 recruitment in nuclear foci confirmed the reduced chromatin localisation of RPA in the WS^E84A^ cells and evidenced a significant decrease in the fraction of RPA32 phosphorylated at S33 (Supplementary Figure S8A, B).

RAD51 participates in multiple pathways (26,27) and chromatin recruitment may reflect such pleiotropy of roles. To further verify the increased recruitment of RAD51 in cells expressing the exo-dead WRN, we performed a quantitative immunofluorescence analysis of the RAD51 foci, which reflects formation of RAD51 nucleofilaments (Figure 2B). Consistent with biochemical data, loss of WRN exonuclease increased recruitment of RAD51 in foci (Figure 2B). Moreover, CPT treatment led to a further enhancement of RAD51 foci in WRN exonuclease-deficient cells compared with the wild-type (Figure 2B). Increased recruitment of RAD51 in chromatin was also observed when the exo-dead WRN was added-back to doxycycline-inducible shWRN cells but not in cells complemented with the wild-type WRN (Supplementary Figure S9). The opposed behavior of RAD51 and RPA chromatin association in WS^E84A^ cells was specific for a low-dose of CPT since it was not detected in response to HU or micromolar CPT (Supplementary Figure S10A). Most notably, BRCA2 depletion abrogated the recruitment in chromatin of RAD51 in WS^E84A^ cells (Supplementary Figure S10B).

**Figure 3.**
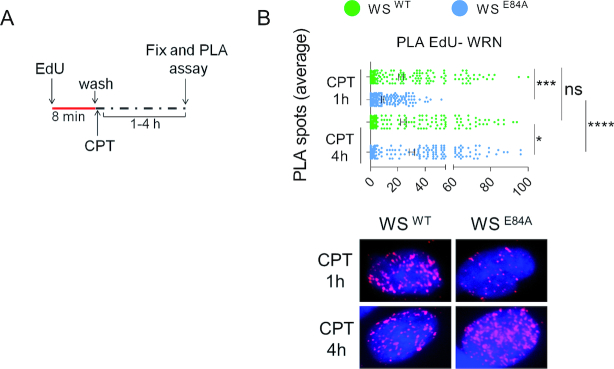
WRN recruitment at CPT-perturbed replication forks is delayed in WS^E84A^ cells. (**A**) Analysis of WRN fork recruitment by *in situ* EdU-PLA. Cells were treated as indicated in the schemes. (**B**) The graph shows the number of PLA spots per cell. Values are presented as means ± SE (ns, not significant; *P < 0.05; ***P < 0.001; ****P < 0.0001; Mann–Whitney test). Representative images are shown.

The biphasic nature of ssDNA accumulation observed in the absence of WRN exonuclease activity in cells treated with a low-dose of CPT prompted us to investigate on the kinetics of replication fork recruitment of the mutant WRN protein by the EdU-PLA technique (SIRF; (28,29)). After a pulse-labeling of nascent DNA with EdU, wild-type cells or cells expressing the exo-dead WRN, were treated with CPT and tested for the association of WRN with EdU-labeled nascent DNA by proximity-ligation assay *in situ* (Figure 3A). The catalytically-active WRN showed a noticeable fork recruitment already at 1 h of CPT treatment, which increased further and significantly at 4 h (Figure 3B). As expected by our previous data (12), the exo-dead WRN was less fork-associated than the wild-type at the early time-point but surprisingly increased its fork association at 4 h reaching the wild-type value (Figure 3B).

These results suggest that, in the absence of the WRN exonuclease, RAD51 takes over RPA and the normal mechanism handling CPT-perturbed replication forks. Moreover, our results also indicate that recruitment of exo-dead WRN at perturbed forks is delayed early but is enhanced at 4h of treatment when the level of nascent ssDNA elevates accordingly.

### Loss of WRN exonuclease stimulates the persistence of under-replicated DNA and RAD51 accumulation during recovery from low-dose of CPT

**Figure 4.**
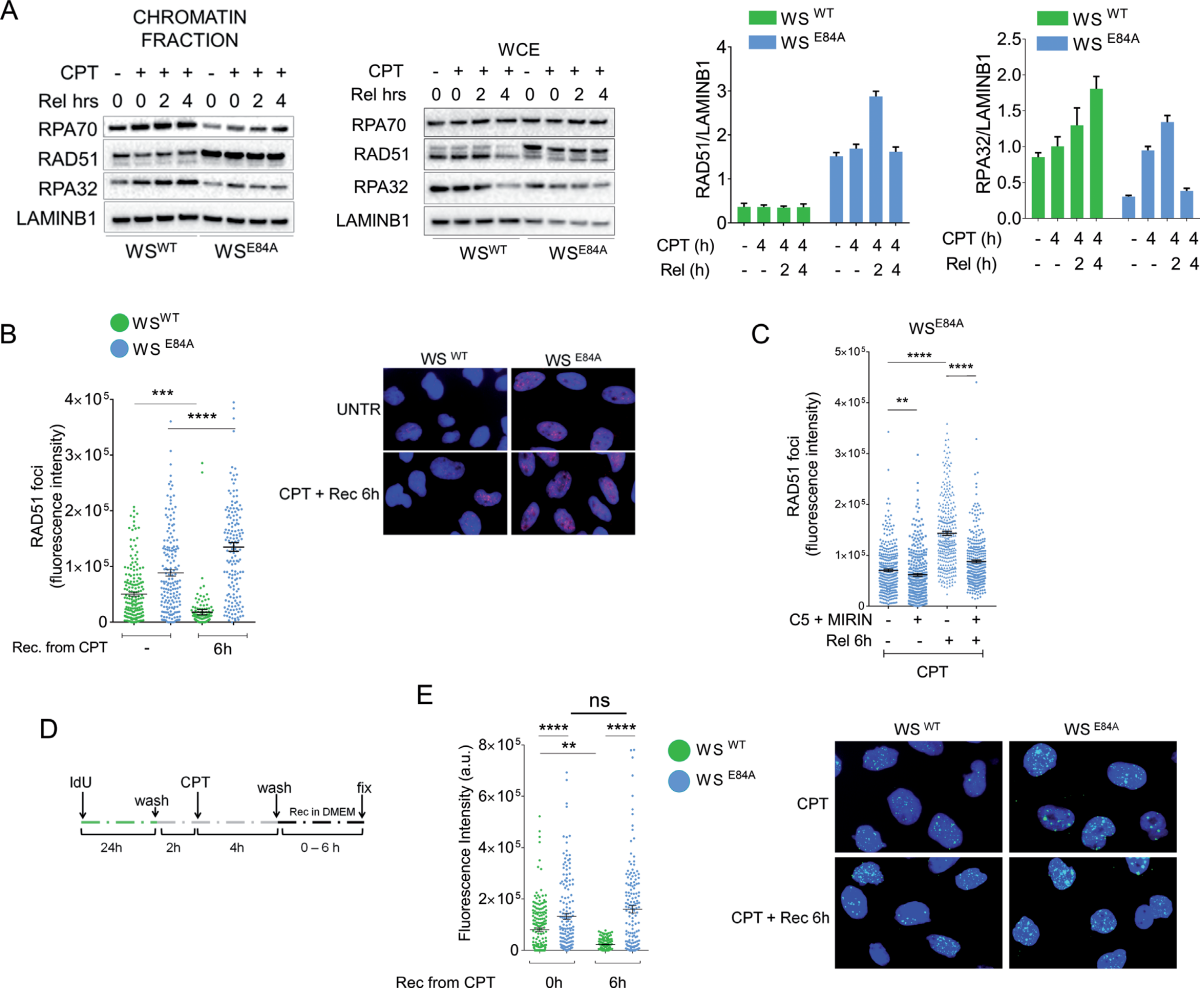
RAD51 recruitment persisted during recovery from CPT in the absence of the WRN exonuclease. (**A**) WB analysis of chromatin association of RAD51, RPA70 and RPA32 in wild-type (WS^WT^) and in cells expressing the exo-dead mutant form of WRN (WS^E84A^). Cells were treated or not with 50 nM CPT for 4h followed by recovery as indicated. LaminB1 was used as loading control. The blot is representative of three replicates. The graphs show the quantification of the amount of RAD51 or RPA32 normalized against LaminB1 (mean ± SE). (**B**) Quantitative immunofluorescence analysis of RAD51 foci in WS^WT^ and WS^E84A^ cells. Cells were treated with CPT 50nM for 4 h and recovered or not as indicated. Graph shows the intensity of RAD51 immunostaining for each cell with scorable foci (*n* > 3). Values are presented as means ± SE (***P < 0.001; **** *P* < 0.0001; Mann–Whitney test). Representative images are shown. (**C**) Quantitative immunofluorescence analysis of RAD51 foci in WS^WT^ and WS^E84A^ cells pre-treated with the nuclease inhibitors. Cells were pre-treated with the indicated inhibitors prior to be challenged with CPT 50nM for 4h and recovered or not as indicated. Graph shows the intensity of RAD51 immunostaining for each cell with scorable foci (*n* > 3). Values are presented as means ± SE (**P < 0.01; ****P < 0.0001; Mann–Whitney test). (D, E) analysis of parental ssDNA. Parental DNA was labeled with IdU as indicated in the experimental scheme (**D**). (**E**) The graph shows the amount of parental ssDNA calculated as mean intensity of IdU fluorescence measured from two independent experiments (*n* = 200), data are presented as mean ± SE. Statistical analysis was performed by the Mann–Whitney test (**P < 0.01; **** *P* < 0.0001). Representative images are shown.

To determine if the increased RAD51 recruitment could correlate with recovery from replication fork perturbation, we analyzed RAD51 chromatin levels after CPT withdrawal. To this end, we treated cells with CPT and allowed them to recover for 2 and 4 h before analyzing chromatin fractions. As shown in Figure 4A, while RAD51 chromatin levels were consistently low during recovery in wild-type cells, they were still elevated in WRN exonuclease-deficient cells. In contrast, the amount of chromatin-bound RPA32 and RPA70, two subunits of trimeric RPA, was low in WS^E84A^ cells during recovery while it increased greatly in cells expressing the wild-type WRN (Figure 4A). To confirm this result, we performed quantitative immunofluorescence analysis of the formation of RAD51 foci. A reduction of RAD51 foci was observed during recovery from CPT in wild-type cells while the number of RAD51 foci increased in WRN exonuclease-deficient cells (Figure 4B).

Inhibition of MRE11 and DNA2 restores wild-type levels of nascent ssDNA in WRN exonuclease-deficient cells (Figure 1C). To determine if the persistence of chromatin-bound RAD51 in WS^E84A^ cells during recovery correlates with the accumulation of nascent ssDNA occurring during treatment, we analyzed RAD51 focus-forming activity in cells exposed to CPT and to the two nuclease inhibitors Mirin and C5. As expected, RAD51 recruitment in foci was elevated in WRN exonuclease-deficient cells after treatment and even more during recovery (Figure 4C). Interestingly, however, treatment with C5 and Mirin before CPT significantly reduced the persistence of RAD51 foci in WS^E84A^ cells during recovery (Figure 4C).

The elevated levels of RAD51 during recovery might be related to the presence of under-replicated DNA that engages recombination to be repaired or fully replicated (30). Thus, we analyzed whether cells recovering from CPT treatment presented under-replicated regions of DNA. Since under-replication is expected to leave regions of ssDNA behind perturbed forks in the parental strand (parental gaps), we performed native ssDNA assay after a 24 h treatment with IdU to label all parental DNA (Figure 4D). As shown in Figure 4E, little parental ssDNA was detectable in wild-type cells after treatment and its amount decreased substantially during recovery. In WS^E84A^ cells, however, parental ssDNA was increased at the end of treatment and remained elevated during recovery (Figure 4E). Interestingly, the increased amount of parental ssDNA paralleled that of RAD51 foci, suggesting that RAD51 may be recruited to deal with under-replicated regions. Both phenotypes observed in WS^E84A^ cells during recovery were recapitulated in the doxycycline-inducible shWRN cells after complementation with the exo-dead WRN (Supplementary Figure S11A–C). Of note, persistence of parental gaps was not related to repair of the poisoned CPT since it was not affected by depletion of TDP1 (Supplementary Figure S12A, B).

Collectively, these results indicate that loss of WRN exonuclease results in persistence of parental gaps DNA during recovery, which correlates to increased RAD51 recruitment. Furthermore, our data suggest that persistence of parental gaps and increased RAD51 during recovery is a consequence of the late accumulation of nascent ssDNA.

### Loss of WRN exonuclease reduces activation of CHK1

**Figure 5.**
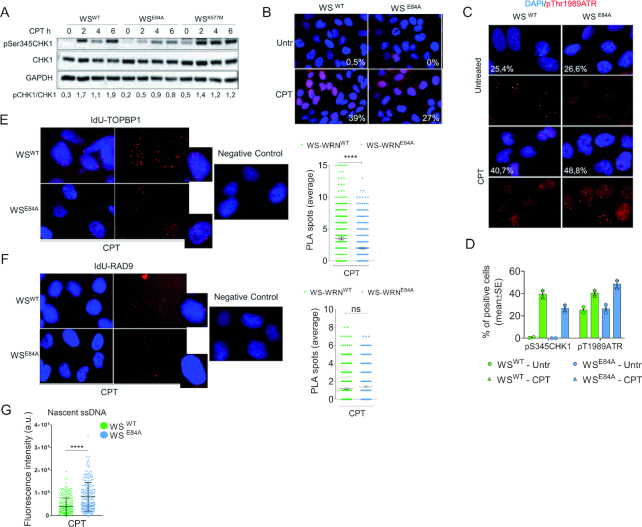
Loss of WRN exonuclease activity affects phosphorylation of CHK1. (**A**) WB analysis of CHK1 phosphorylation at S345 in wild-type (WS^WT^) and in cells expressing the exo-dead mutant form of WRN (WS^E84A^) or the helicase-dead form (WS^K577M^). Cells were treated or not with 50nM CPT as indicated. Total CHK1 and GAPDH were used as loading controls. The blot is representative of three replicates. Below is reported quantification of pS345CHK1 phosphorylation normalized against total CHK1. (**B**) Immunofluorescence analysis of pS345CHK1 in WS^WT^ and WS^E84A^ cells treated with CPT 50nM for 4 h. Numbers in insets represent the mean percentage of pS345CHK1-positive nuclei (*n* = 2). (**C**) Immunofluorescence analysis of pT1989ATR in WS^WT^ and WS^E84A^ treated with CPT 50nM for 4 h. Numbers in insets represent the mean percentage of pS345CHK1-positive nuclei (*n* = 2). (**D**) Quantification of the anti-pS345 and pT1989ATR IF. (E-F) Analysis of TopBP1 or RAD9 recruitment at nascent ssDNA by PLA. Nascent strand was labeled with IdU for 15 min before cells were treated with 50nM CPT for 4 h. PLA was performed under native conditions using anti-IdU to detect nascent ssDNA and anti-TopBP1 or RAD9 to detect the protein. Negative controls are from samples processed with anti-IdU only. The graphs show the number of PLA spots in each nucleus (*n* = 300 from three independent replicates). Statistical analysis was performed by the Mann–Whitney test (ns = not significant; ****P < 0.0001). Representative images are shown. (G) Duplicated samples from D and E were analyzed for the presence of nascent ssDNA by native anti-IdU detection only. The graph shows the mean intensity of IdU fluorescence measured from two independent experiments (*n* = 200), data are presented as mean ±SE. Statistical analysis was performed by the Mann–Whitney test (***P < 0.001).

Our data indicate that loss of the WRN exonuclease results in accumulation of ssDNA and RAD51 accompanied by a concomitant decrease of RPA and pS33RPA32 in chromatin. Since RPA-coated ssDNA is required for checkpoint signaling upon replication fork perturbation, we investigated whether the functionality of the WRN exonuclease might also affect activation of the replication checkpoint in response to nanomolar concentration of CPT. As a readout of the activation of the ATR-dependent checkpoint response, we analyzed phosphorylation of CHK1 at S345 by western blotting. In wild-type cells, treatment with a nanomolar dose of CPT induced a time-dependent phosphorylation of CHK1 which is also readily observed in cells expressing the helicase-dead form of WRN (WS^K577M^) (Figure 5A). In contrast, CPT-induced phosphorylation of CHK1 was reduced in cells expressing the exonuclease-dead mutant of WRN, and this phenotype was more evident at 4 and 6h of treatment (Figure 5A). The requirement of the WRN exonuclease for correct CHK1 phosphorylation was specific for the low-dose CPT treatment as it was not observed after 5μM of CPT (Supplementary Figure S13A).

To confirm that loss of WRN exonuclease affected CHK1 phosphorylation by an independent assay, we monitored the status of S345 of CHK1 by immunofluorescence. As shown in Figure 5B and D, a reduced phosphorylation of CHK1 at S345 was readily detected also by immunofluorescence in WRN exonuclease-deficient cells.

Next, we wanted to analyze whether decreased activation of CHK1 correlated with reduced activation of ATR-dependent signaling. To assess activation of ATR, we monitored phosphorylation of the activating site T1989 by immunofluorescence. Despite the defective phosphorylation of CHK1, ATR was phosphorylated similarly in wild-type cells and in cells expressing the exonuclease-dead form of WRN (Figure 5C, D). Since loss of WRN exonuclease affects recruitment of RPA but did not affect activation of ATR, we analyzed whether it could influence recruitment of other factors modulating ATR-checkpoint function. As loss of WRN exonuclease leads to accumulation of nascent ssDNA at 4h of CPT (Figure 1A), we analyzed the presence of TopBP1 and RAD9, which associates with TopBP1 (31,32), specifically at nascent ssDNA by our recently described IdU-PLA assay (12). In parallel, we evaluated the presence of the total amount of ssDNA by IdU assay. Association of TopBP1 or RAD9 with nascent ssDNA was not detected under untreated conditions (data not shown), however, treatment with 50nM CPT for 4h resulted in recruitment of both factors at nascent ssDNA in wild-type cells (Figure 5E, F). Of note, loss of WRN exonuclease reduced the recruitment of TopBP1 but not that of RAD9 at the nascent ssDNA (Figure 5E, F) although in these conditions the IdU assay detected 2-times more ssDNA (Figure 5G).

**Figure 6.**
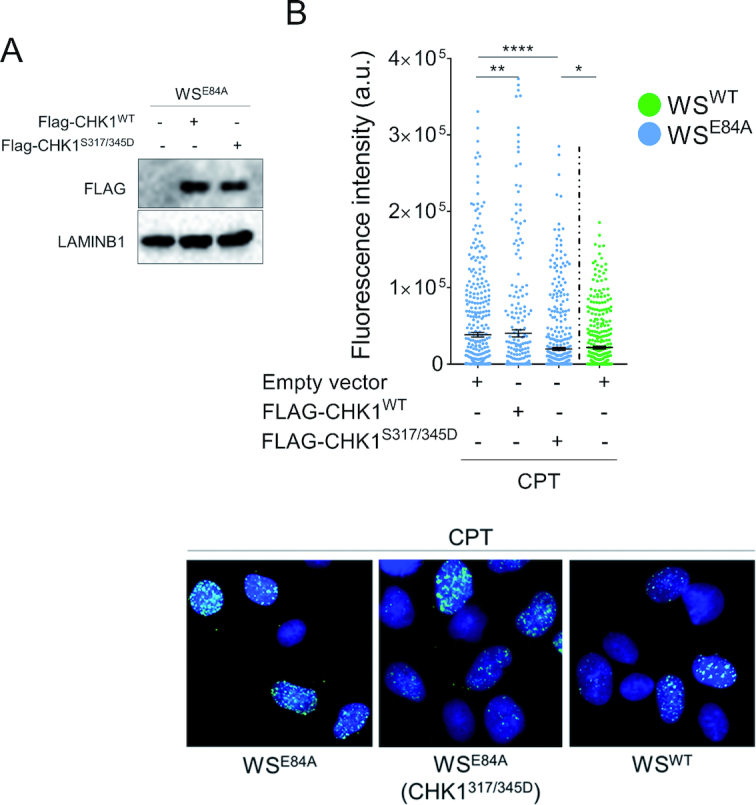
Expression of a phosphomimic CHK1 mutant restores wild-type levels of nascent ssDNA in WRN exonuclease-deficient cells. (**A**) WB analysis of FLAG-CHK1^WT^ and FLAG-CHK1^S317/345D^ expression in WS^E84A^ cells. (**B**) Evaluation of nascent ssDNA formation. Cells treated with 50nM CPT for 4 h were analyzed for the presence of nascent ssDNA by native anti-IdU detection. The graph shows the mean intensity of IdU fluorescence measured from two independent experiments (*n* = 200), data are presented as mean ± SE. Statistical analysis was performed by the Mann–Whitney test (*P < 0.05; ***P < 0.001; ****P < 0.0001). Representative images are shown.

Loss of the WRN exonuclease leads to accumulation of nascent ssDNA, which is targeted by RAD51 and not by checkpoint factors, possibly resulting in reduced CHK1 phosphorylation. Thus, we investigated whether pre-treatment with the RAD51 inhibitor B02 or could re-establish a normal CHK1 activation in WRN exonuclease-deficient cells (Supplementary Figure S13B). Of note, inhibition of RAD51 further decreased the phosphorylation of CHK1 regardless the way it was added (Supplementary Figure S13B). This was surprising but prompted us to evaluate whether reduced CHK1 activation could be implicated in the enhanced accumulation of nascent ssDNA. To test this potential feedback effect, we over-expressed the S317/S345D CHK1 phosphomimetic mutant (22) in WS^E84A^ cells before evaluating the formation of nascent ssDNA by the IdU assay (Figure 6A). As shown in Figure 6B, the phosphomimetic CHK1 and the wild-type form were efficiently over-expressed in the cells, however, only the phosphomimetic CHK1 mutant was able to substantially reduce the amount of nascent ssDNA in WRN exonuclease-deficient cells restoring the wild-type levels, while overexpression of the wild-type increased further the amount of nascent ssDNA.

Altogether, our results show that loss of WRN exonuclease activity affects proper activation of CHK1 in response to a low-dose of CPT and that reduced phosphorylation of CHK1 probably correlates with reduced recruitment of checkpoint factors at ssDNA. They also suggest that reduced CHK1 phosphorylation contributes to the accumulation of ssDNA in the nascent strand, possibly as part of a positive feedback loop.

### WRN exonuclease-deficient cells need the mitotic function of MUS81 to counteract mitotic aberration and mis-segregation

**Figure 7.**
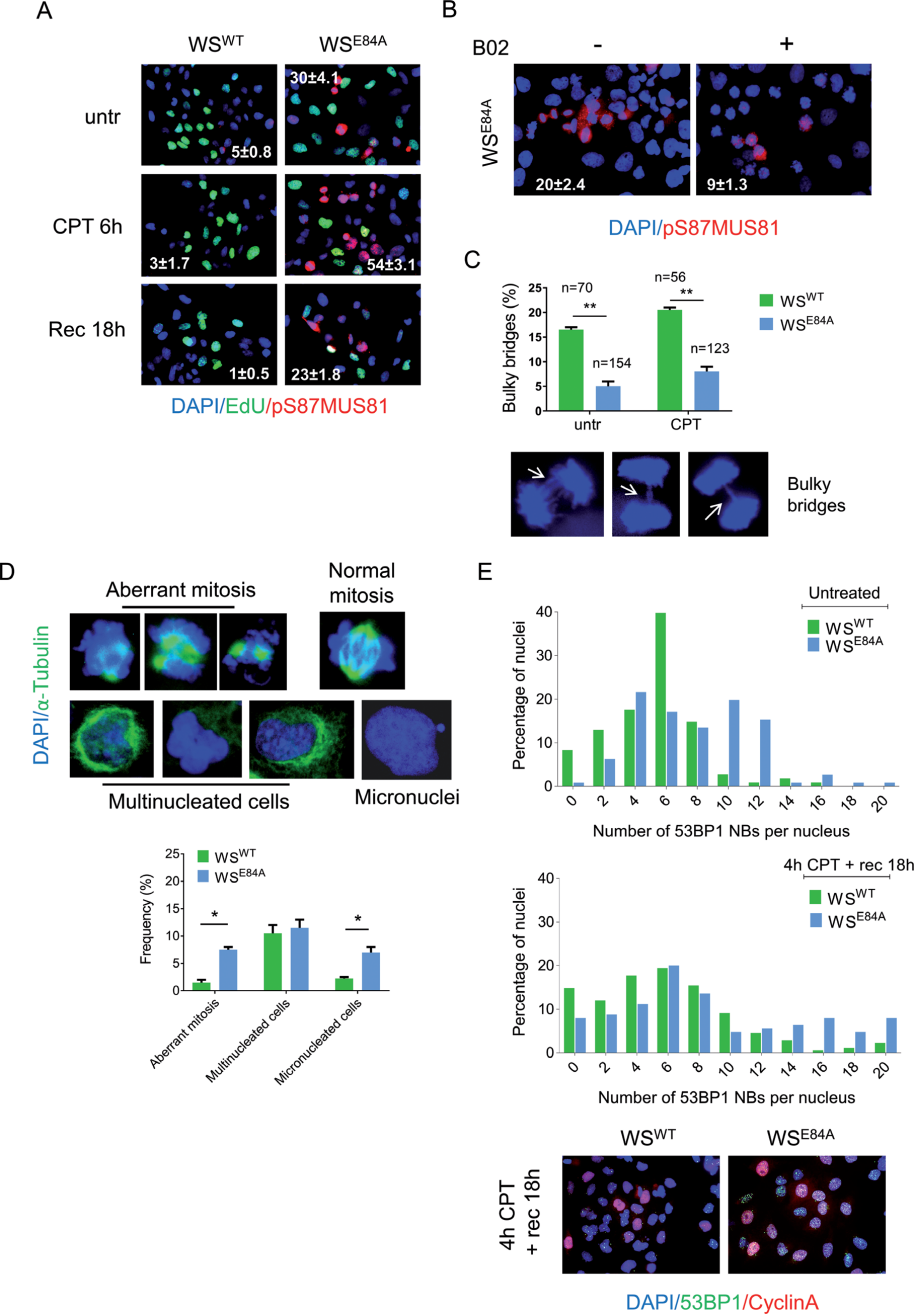
WRN exonuclease-deficient cells show enhanced MUS81 phosphorylation on S87 and mitotic defects. (**A**) Anti-pS87MUS81 immunofluorescence staining (red) was performed in wild-type and WRN exonuclease-dead expressing cells. The S-phase cells (green) were revealed with short EdU pulse followed by Click-IT reaction. Nuclei were depicted with DAPI staining (blue). The mean frequency (±SE; *n* = 3) of pS87-MUS81-positive nuclei are indicated in the representative images. (**B**) WS cells expressing the WS^E84A^ mutant were treated with CPT for 4h and then released in fresh medium for 18 h. The RAD51 inhibitor B02 was added with CPT and during the recovery. The frequency (±SE; *n* = 2) of pS87-MUS81-positive nuclei are indicated as percentage in the representative images. (**C**) The graph shows the mean percentage ± SE of bulky anaphase bridges analyzed in untreated and CPT-treated cells expressing WRN wild-type and the exonuclease-deficient mutant. The number of anaphases counted for each experimental point are indicated above as *n*. Randomly-selected representative anaphases with bridges are shown. (**D**) Representative images of mitotic aberrations analyzed in α-Tubulin (green) and DAPI-stained cells are shown above the graph indicating the frequency of each event after treatment with 50nM of CPT for 4h followed by a 18 h recovery. Data are presented as mean ± SE. Statistical analysis was performed by the ANOVA test (*P < 0.05). (**E**) Analysis of 53BP1 NBs. Cells were either untreated or treated with 50nM CPT as indicated. Samples were subjected to immunofluorescence using anti-53BP1 and anti-Cyclin A to evaluate 53BP1 fluorescence only in G1 cells (Cyclin A-negative). Graphs show the frequency of each class of nuclei in two independent replicates. Representative images from CPT-treated samples are shown.

Inaccurate processing of perturbed replication forks, under-replicated DNA and elevated RAD51 levels observed in the absence of WRN exonuclease could threaten mitosis because of DNA interlinking as reported in BRCA2-deficient cells (33). Mitotic resolution of DNA interlinked intermediates involves the MUS81/EME1 complex (34,35). Thus, we evaluated whether WRN exonuclease-deficient cells accumulated active MUS81 in mitosis by performing immunofluorescence with an antibody directed against the pS87-MUS81, which we have demonstrated to mark the active MUS81/SLX4 complex (36). In wild-type cells, little MUS81 phosphorylation at S87 was detectable under unperturbed cell growth or in response to low-dose of CPT (Figure 7A). In contrast, many pS87-MUS81-positive nuclei were observed in cells expressing the exonuclease-dead WRN already in unperturbed conditions (Figure 7A). Notably, in WRN exonuclease-deficient cells, pS87-MUS81 levels were further enhanced by CPT and remained elevated also after recovery (Figure 7A). Interestingly, and consistent with our previous results (36), phosphorylation of MUS81 never occurred in EdU-labeled S-phase cells (Figure 7A). To determine if under-replication and enhanced RAD51 recruitment correlates with elevated MUS81 activation, we asked whether treatment with B02 (37) reverted the pS87-MUS81 levels in WRN exonuclease-deficient cells. As shown in Figure 7B, inhibition of RAD51 greatly reduced pS87-MUS81-positive nuclei after recovery from CPT in the absence of the WRN exonuclease activity.

Enhanced activation of MUS81 in mitosis might be indicative of persistence of unresolved DNA intermediates in WS^E84A^ cells, which could induce mitotic abnormalities or segregation defects. To assess if loss of WRN exonuclease could result in segregation defects, we first analyzed the presence of bulky anaphase bridges in DAPI-stained cells (Figure 7C). Interestingly, anaphase cells were highly enriched in WRN exonuclease-deficient cells (Figure 7C; see numbers above the bars), suggesting that these cells may experience a delayed exit from anaphase. However, the number of anaphases with bridges was very low in WRN exonuclease-deficient cells as compared to the wild-type (Figure 7C). Since delayed exit from anaphase might derive from mitotic defects and could result in post-mitotic abnormalities, we decided to evaluate the presence of aberrant mitoses, multinucleated cells and micronuclei. As shown in Figure 7D, we found that both aberrant mitosis and cells with micronuclei were increased in WS^E84A^ cells. In contrast, no difference in multinucleated cells was found between cells expressing wild-type or exo-dead WRN (Figure 7D).

The presence of under-replicated DNA or the persistence of unresolved DNA intermediates in G2/M triggers the formation of 53BP1 NBs in the subsequent G1 phase (38). Since WRN exonuclease-deficient cells showed persistence of under-replicated DNA, we investigated whether they accumulated 53BP1 NBs. To this end, we performed immunofluorescence against 53BP1 and CyclinA in cells recovering from 4h of treatment with CPT and scored the number of 53BP1 NBs-positive cells in the CyclinA-negative population (i.e. G1 cells). As shown in Figure 7E, the number of 53BP1 NBs in WS^E84A^ cells was higher than in wild-type cells even in untreated conditions. Treatment with CPT enhanced the number of 53BP1 NBs in wild-type and in WRN exonuclease-deficient cells; however, the increase was substantially higher in cells expressing the exo-dead WRN (Figure 7E and Supplementary Figure S14).

Notably, inhibition of RAD51 in WRN exonuclease-deficient cells resulted in persistent accumulation of mitotic cells in pro-metaphase (Supplementary Figure S15). This result suggested that loss of RAD51 undermines correct mitotic progression in WRN-exonuclease-deficient cells but prevented assessment of any correlation between enhanced engagement of RAD51 and mitotic defects or formation of 53BP1 NBs.

**Figure 8.**
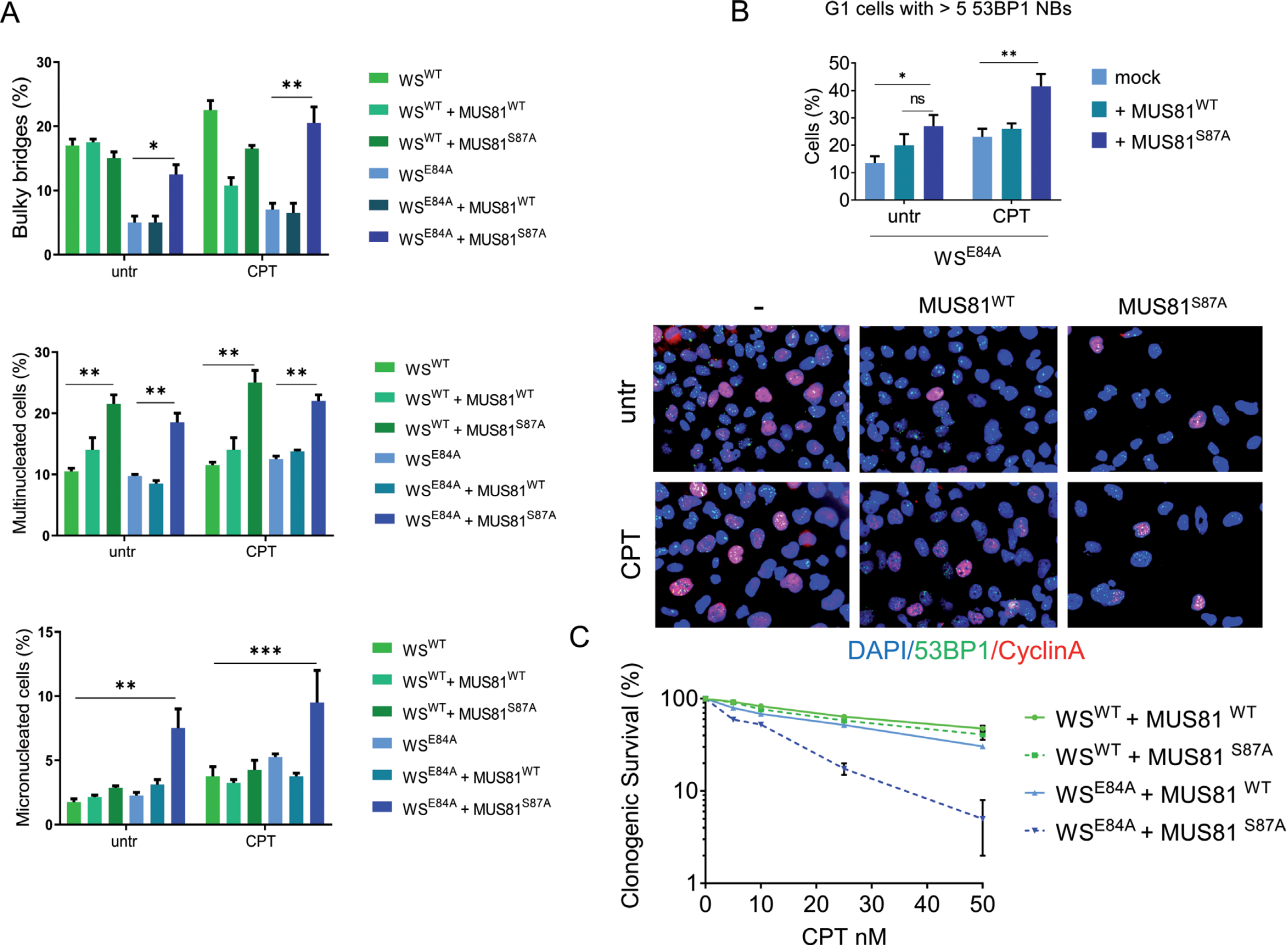
MUS81^S87A^ mutant overexpression aggravates the mitotic phenotypes of WRN exonuclease-deficient cells. (**A**) Bulky bridges, multinucleated and micro-nucleated cells were analyzed with or without MUS81^S87A^ mutant overexpression, in WRN wild-type and exonuclease-deficient cells. The graph represents the frequency of the aberration analyzed in two independent experiments ± SE. (**B**) Analysis of 53BP1 NBs formation in Cyclin A-negative cells. Representative images of fluorescence cells stained with anti-53BP1 (green) and Cyclin A (red). Nuclear DNA was counterstained with DAPI (blue). For each point at least 300 nuclei were counted and cells with >5 53BP1 NBs were considered as positive. The graph shows the quantification of 53BP1-positive G1 cells. (**C**) Clonogenic assay in cells treated with low-doses of CPT. Cells were exposed to different doses of CPT for 18h, re-plated at low density and survival evaluated as percentages of colonises normalized against the untreated. Statistical analyses in A-C were performed by ANOVA test (*P < 0.05; ** *P* < 0.01).

In WRN exonuclease-deficient cells engagement of RAD51 is functionally related to elevated levels of S87-MUS81 phosphorylation (Figure 7B). Thus, we next analyzed whether inactivation of the mitotic function of MUS81 by overexpression of the unphosphorylable S87A-MUS81 mutant (36) in these cells could aggravate the mitotic defects. Interestingly, over-expression of the S87A-MUS81 mutant in wild-type cells did not affect the percentage of anaphase bridges and micronuclei, while it increased the number of multinucleated cells (Figure 8A). In sharp contrast, expression of the S87A-MUS81 mutant substantially aggravated the mitotic defects in WRN exonuclease-deficient cells (Figure 7A). Indeed, expression of the S87A-MUS81 protein increased the number of anaphase bridges and micronucleated cells. Similarly, expression of the S87A-MUS81 mutant enhanced the presence of 53BP1 NBs in WS^E84A^ cells (Figure 8B).

To further assess the biological significance of the mitotic MUS81 hyperactivation observed in the absence of the WRN exonuclease, we evaluated the sensitivity to nanomolar doses of CPT by clonogenic survival. As shown in Figure 8C, WRN exonuclease-deficient cells were slightly more sensitive to CPT then wild-type cells. Overexpression of the wild-type MUS81 resulted in a mild increase in sensitivity in wild-type cells but not in cells expressing the exo-dead WRN (Figure 8C). In contrast, overexpression of the S87A-MUS81 substantially increased the sensitivity of WRN exonuclease-deficient cells to CPT (Figure 8C).

Altogether, our data indicate that the enhanced engagement of RAD51 associated to loss of WRN exonuclease requires the increased activation of the MUS81 complex in mitosis. Therefore, expression of a MUS81 mutant that disables mitotic activation of the MUS81/EME1 complex increases mitotic abnormalities and sensitivity to CPT of WRN exonuclease-deficient cells. Thus, in the absence of the WRN exonuclease, hyperactivation of the MUS81 complex functions as a fail-safe system that maintains mitotic abnormalities at low levels, allowing survival.

## DISCUSSION

In recent years, an increasing interest arose around alternative mechanisms of fork processing and fork degradation since they correlate to the chemosensitivity of cells that are deficient for the primary pathway represented by BRCA1/2-RAD51 (16,18). Most of these studies focused on the early events occurring in the absence of BRCA1 or BRCA2, but few of them investigated mechanisms involved during recovery from replication stress (33,39). Furthermore, loss of BRCA1 or BRCA2 affects recombination as well as fork protection and this prevents the investigation of the role of recombination for the recovery of replication forks undergoing degradation. Recently, we reported that the WRN exonuclease activity protects against fork degradation when cells are treated with clinically-relevant doses of CPT (12). Here, we used WRN exonuclease-deficient cells as a model to assess what happens at destabilized perturbed forks when treatment with nanomolar doses of CPT is prolonged. We find that, in the absence of the WRN exonuclease, nascent strands undergo continuous degradation that produces accumulation of ssDNA. This late accumulation of ssDNA follows its disappearance at early time points because of the activities of MRE11 and EXO1 (12). Interestingly, the late wave of ssDNA at perturbed forks is only minimally affected by inactivation of each single exonuclease acting at perturbed forks, and is reduced only when EXO1 is depleted or when MRE11 and DNA2 are inhibited. While most of the degradation observed in cells deficient of BRCA1/2 or other factors assisting RAD51 seems to involve MRE11-EXO1 (40–43), our data suggest that multiple nucleases take over at forks destabilized by the absence of WRN exonuclease with time. Treatment with nanomolar doses of CPT does not induce DSBs unless treatment is prolonged (13,14). Interestingly, loss of the WRN exonuclease makes cells resistant to the induction of DSBs. Induction of DSBs in response to nanomolar doses of CPT has been correlated with activation of RECQ1 possibly to promote restart of those forks that failed to be processed otherwise (14). Loss of the ability to induce DSBs at forks would be consistent with engagement of a distinct fork recovery mechanism in cells expressing the exo-dead WRN protein. Of note, RECQ1 PARylation, which regulates RECQ1 function at reversed forks after CPT (14), is almost absent in WRN exonuclease-deficient cells (12). The concomitant absence of RECQ1-regulatory modification and of its consequences (i.e. DSBs) strongly support a pathway switch at CPT-perturbed forks.

Consistent with this pathway switch, inhibition of MRE11 in WRN exonuclease-deficient cells restores DSBs after prolonged treatment with a low-dose of CPT. This suggests that formation of DSBs occurs downstream of pathological fork processing after takeover of other nucleases or that the increase in nascent ssDNA observed after MRE11 inhibition in WRN exonuclease-deficient cells derives from end-resection at DSBs. Given that each of the nucleases has been implicated in end-resection, the former possibility is more likely.

Interestingly, RAD51 is strongly accumulated in the absence of the WRN exonuclease and persists during recovery from treatment. Of note, the enhanced recruitment of RAD51 in chromatin parallels the reduced level of RPA. Since binding of RPA is a pre-requisite for the binding of RAD51, it is likely the RAD51-RPA exchange mediated by BRCA2 occurs more efficiently in cells expressing the exo-dead WRN. An elevated engagement of RAD51 in the absence of the WRN exonuclease has been reported in *Drosophila* (44), suggesting that the role of WRN exonuclease at perturbed forks is conserved. Similarly, unscheduled exonuclease-mediated processing of perturbed forks in yeast has been recently shown to engage a RAD51-mediated pathway (45). Furthermore, parental ssDNA, a readout of template gaps, also accumulates in the absence of WRN exonuclease. RAD51 binds to ssDNA and initiates recombination (20,30). The concomitant accumulation of ssDNA and elevated recruitment of RAD51 in the absence of the WRN exonuclease would be consistent with the engagement of gaps left behind inactivated forks in a template-switch mode of replication recovery, as shown after DNA damage in *Xenopus* egg extracts (26). Since RAD51 and WRN have been proposed to cooperate during recovery from fork arrest (46), it is reasonable that loss of WRN function also compromises the normal activity of RAD51 at fork.

Loss of WRN exonuclease results in a mild defect in the activation of CHK1. Activation of the ATR-dependent checkpoint requires formation of ssDNA (47–49). In the absence of the WRN exonuclease ssDNA accumulates but is hijacked by RAD51 and it is not completely free for the binding of checkpoint factors. Indeed, in WRN exonuclease-deficient cells, TopBP1 and its binding factor RAD9 are not more highly associated with ssDNA as compared with the wild-type. Notably, phosphorylation of ATR, a readout of its activation, is indistinguishable from the wild-type. It suggests that ATR also gets activated independently from ssDNA. Alternatively, a hyperactivation of ATR could be prevented by RAD51 through sequestering of ssDNA. Indeed, overexpression of RAD51 has been shown to affect checkpoint activation (50). Thus, CHK1 phosphorylation is probably dampened because of a faster or more efficient RPA-RAD51 exchange that also correlates to reduced recruitment of RAD9 and TopBP1. Notably, bypassing of the CHK1 activation defect by expression of a phosphomimetic CHK1 mutant in WRN exonuclease-deficient cells restores normal levels of ssDNA. This suggests that accumulation of ssDNA is also unleashed by reduced CHK1 activation through a positive feedback loop.

The observed elevated recruitment of RAD51, which is used during recovery in the absence of the WRN exonuclease to deal with under-replicated DNA, also leads to elevated phosphorylation of MUS81 at S87. Phosphorylation of MUS81 at S87 occurs in G2/M and is related to resolution of recombination intermediates (36). Consistently, inhibition of RAD51 reduces S87 phosphorylation in WRN exonuclease-deficient cells. Thus, engagement of RAD51-dependent fork recovery, possibly by template switching at parental gaps, results in an increased number of interlinked intermediates calling for resolution by the MUS81 complex. Our data indicate that activation of MUS81 complex in G2/M is essential to overcome segregation defects arising from excessive RAD51-dependent recombination and support proliferation upon treatment with CPT. Indeed, expression of the unphosphorylable S87A-MUS81 mutant increases abnormal mitosis and sensitizes WRN exonuclease-deficient cells.

Loss of the WRN exonuclease, although resulting in fork degradation, does not induce MUS81 activation in S-phase, which is observed in the absence of BRCA2 (42). However, the persistence of under-replicated DNA and requirement of MUS81 complex activity in G2/M also characteristic of BRCA2-deficient cells (39). Thus, it is tempting to speculate that elevated fork degradation correlates with inability to replicate all the genome. Notably, BRCA2-deficient cells show much more severe mitotic defects (33,39). In WRN exonuclease-deficient cells, mitotic abnormalities are increased by disabling MUS81 function in mitosis but are likely increased also by impairing RAD51 function during recovery, since RAD51 inhibition results in increased accumulation of parental ssDNA and induces a significant mitotic block. As BRCA2 deficiency also interferes with the post-replicative function of RAD51 (26) it is tempting to speculate that the elevated mitotic defects might be the end-result of combined fork deprotection and recombination defects. Indeed, it has been recently demonstrated that mitotic abnormalities in BRCA2-deficient cells are primarily linked to loss of the recombination function of RAD51 (33).

**Figure 9.**
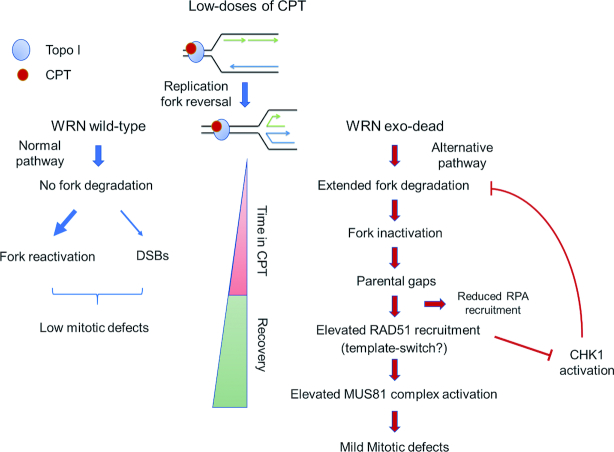
Proposed model of the effect of prolonged treatment with nanomolar CPT doses in absence of WRN exonuclease (see text for details).

Collectively, our data show that WRN exonuclease-deficient cells can be a useful model to investigate the fate of deprotected or destabilized replication forks under a clinically-relevant, specific type of replication stress; and, together with published data, they can be summarized in the model shown in Figure 9. In response to nanomolar CPT, perturbed replication forks rapidly undergo fork reversal (13). In wild-type cells, with time, reversed forks degenerate into DSBs, possibly because of unscheduled RECQ1-mediated fork restoration (14). In the absence of WRN exonuclease, perturbed replication forks undergo a further cycle of degradation of nascent strand by MRE11 and/or DNA2, which leads to ssDNA accumulation and engagement of RAD51. The WRN exonuclease activity might be required to clear stalled Okazaki fragments by promoting their 3’-5’ degradation or to remove D-loop intermediates (51). In the first case, replication fork reversal might be restrained also avoiding excessive, post-reversal, degradation of the nascent strands. In the second case, unscheduled engagement of recombination either from reversed forks or stalled forks might be prevented. Since the two possibilities are not mutually-exclusive, the elevated engagement of recombination seen in the absence of WRN exonuclease may result from a complex phenotype. The accumulation of ssDNA and possibly engagement of RAD51 make perturbed replication forks resistant to DSBs and interfere with checkpoint signaling, resulting in a mild defect in CHK1 activation. In the absence of WRN exonuclease, more perturbed forks become inactivated over-time and RAD51 is required also during recovery from CPT to support repair at template gaps left behind the inactive forks. In WRN exonuclease-deficient cells, engagement of RAD51 during recovery stimulates activation of the MUS81 complex in G2/M and limit mitotic defects and cell death as also occur at difficult-to-replicate regions (52).

As CPT is a chemotherapeutic, our data also indicate that tumors with impaired function of the WRN exonuclease can be sensitized to treatment by genetic or chemical interference with the MUS81 complex in mitosis, which is less relevant for survival in cells expressing the WRN wild-type.

## Supplementary Material

gkz431_Supplemental_Files
