# Supplementary material for: RAD51 and mitotic function of mus81 are essential for recovery from low-dose of camptothecin in the absence of the WRN exonuclease
Source: Nucleic Acids Res. 2019 May 22;47(13):6796–810. doi: 10.1093/nar/gkz431 (PMC6648349; doi:10.1093/nar/gkz431)
Supplement: gkz431_Supplemental_Files [file gkz431_supplemental_files.pdf]

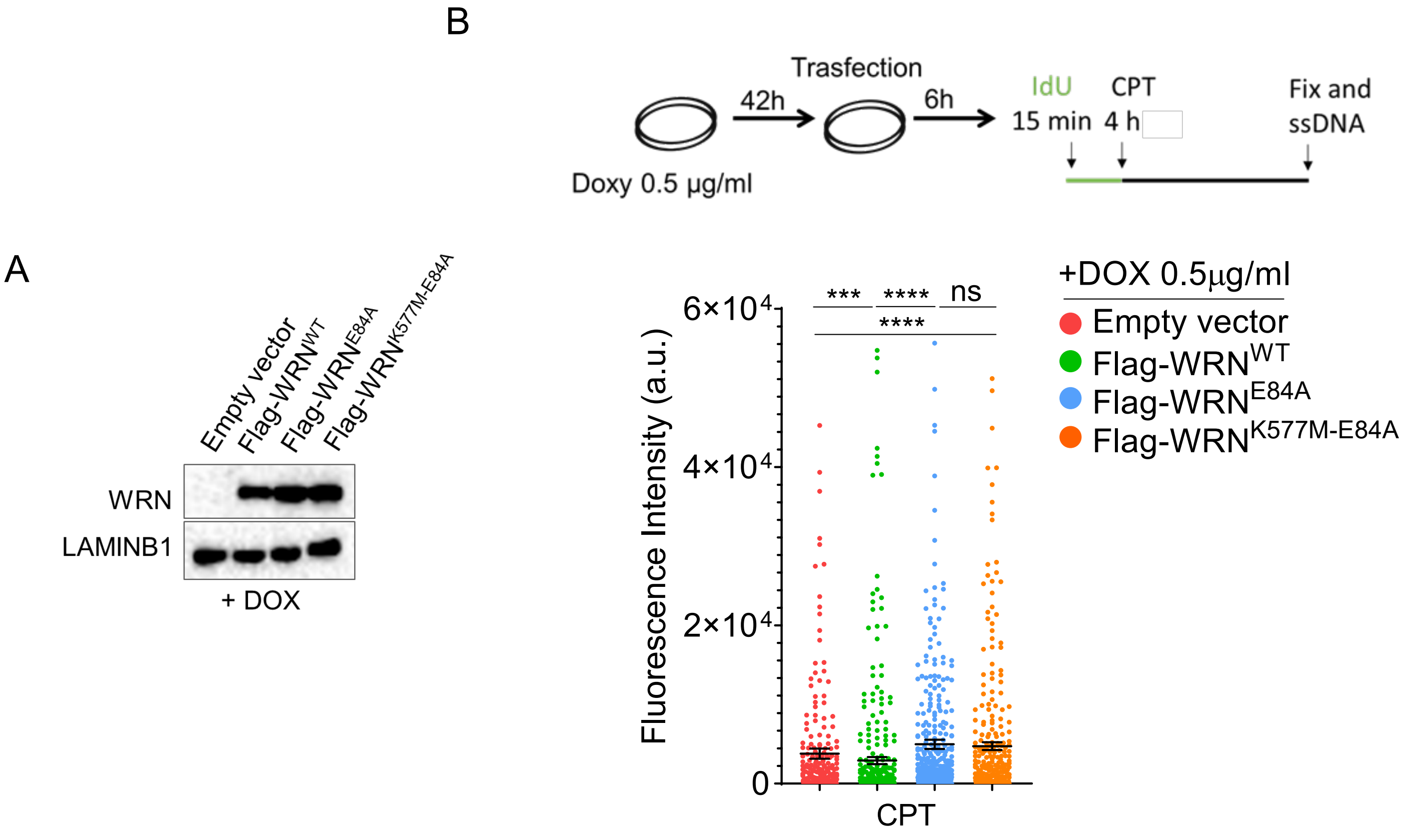

Figure S1. The excessive nascent ssDNA formation is cell line independent and does not correlate with uncopling between the helicase and exonuxlease activities of WRN. (**A**) Western blot shows levels of WRN wild-type (Flag-WRN^{WT}) and its mutants (Flag-WRN^{E84A}, Flag-WRN$^{K577M\text{-}E84A}$) after nucleofection in doxycycline-inducible MRC5-SV40 shWRN cells. Doxycycline was added 42 h before DNA transfection by nucleofection and cells were collected at 48 h. LAMINB1 was used as loading control. (**B**) Evaluation of ssDNA by anti-IdU immunofluorescence under non-denaturing condition was performed as shown in the scheme. Dot plots show the mean intensity of ssDNA staining for single nuclei from cells expressing the wild-type (Flag-WRN^{WT}), the exonuclease dead (Flag-WRN^{E84A}) or the exonuclease-helicase dead (Flag-WRN$^{K577M\text{-}E84A}$) RNAi-resistant form of WRN. Cells were challenged with 50 nM CPT for 4 h. The graph shows the mean intensity of IdU fluorescence measured from two independent experiments (n=300), data are presented as mean ±SE. Statistical analysis was performed by the Mann–Whitney test (ns = not significant; ***P < 0.001; ****P < 0.0001).

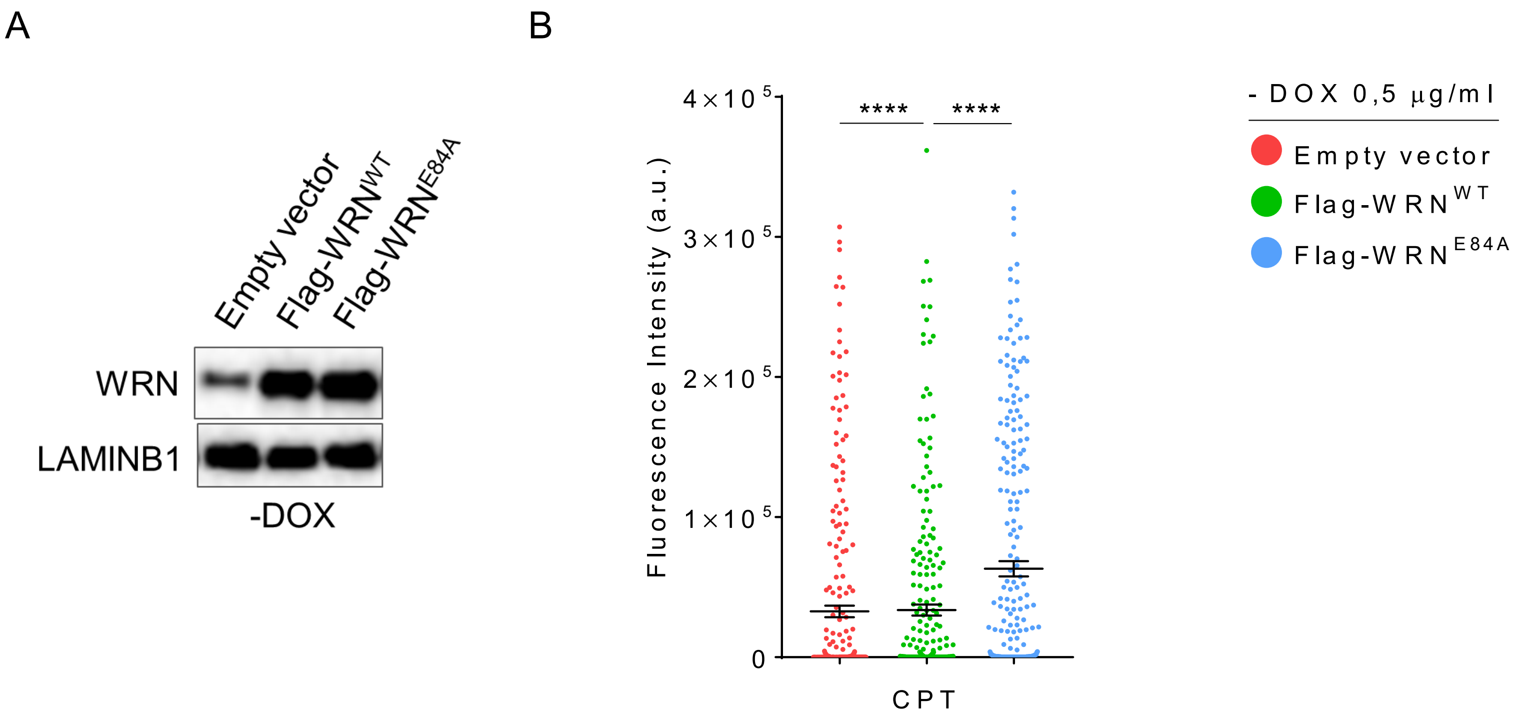

Figure S2. **Nascent ssDNA accumulation in the exo-dead mutant of WRN is not dependent by its overexpression.** (**A**) Western blot shows levels of WRN wild-type (Flag-WRN^{WT}) and exonuclease dead mutant (Flag-WRN^{E84A}) after nucleofection in doxycycline-inducible MRC5-SV40 shWRN cells, without silencing the endogenous protein. (**B**) Evaluation of ssDNA by anti-IdU immunofluorescence under non-denaturing condition was performed 48 h after DNA nucleofection. Nascent DNA was pre-labelled for 15 min with IdU before treatment and labelling remained during treatment with CPT. Dot plots show the mean intensity of ssDNA staining for single nuclei from cells expressing the wild-type (Flag-WRN^{WT}) or the exonuclease dead (Flag-WRN^{E84A}) RNAi-resistant form of WRN. Cells were challenged with 50 nM CPT for 4hrs. The graph shows the mean intensity of IdU fluorescence measured from two independent experiments (n=250), data are presented as mean ±SE. Statistical analysis was performed by the Mann–Whitney test (****$P < 0.0001$).

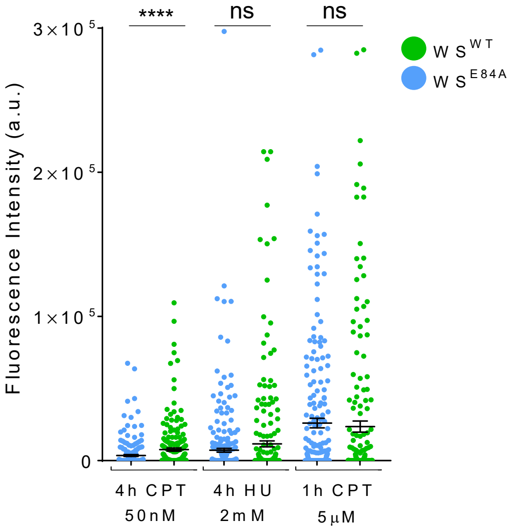

Figure S3. **Difference in the exposure of nascent ssDNA in WS^{E84A} is specific for the CPT low-dose treatment**. To confirm the specificity of the response to a low-dose of CPT as treatment in our model, we treated wild-type (WS^{WT}) and exonuclease-dead (WS^{E84A}) cells with HU or a high-dose of CPT in order to test the aberrant accumulation of nascent ssDNA. Nascent DNA was pre-labelled for 15 min with IdU before treatment and labelling remained during treatment with CPT low-dose, HU or CPT high-dose. The graph shows the mean intensity of IdU fluorescence measured from two independent experiments (n=200), data are presented as mean ±SE. Statistical analysis was performed by the Mann–Whitney test (ns = not significant; ****$P < 0.0001$).

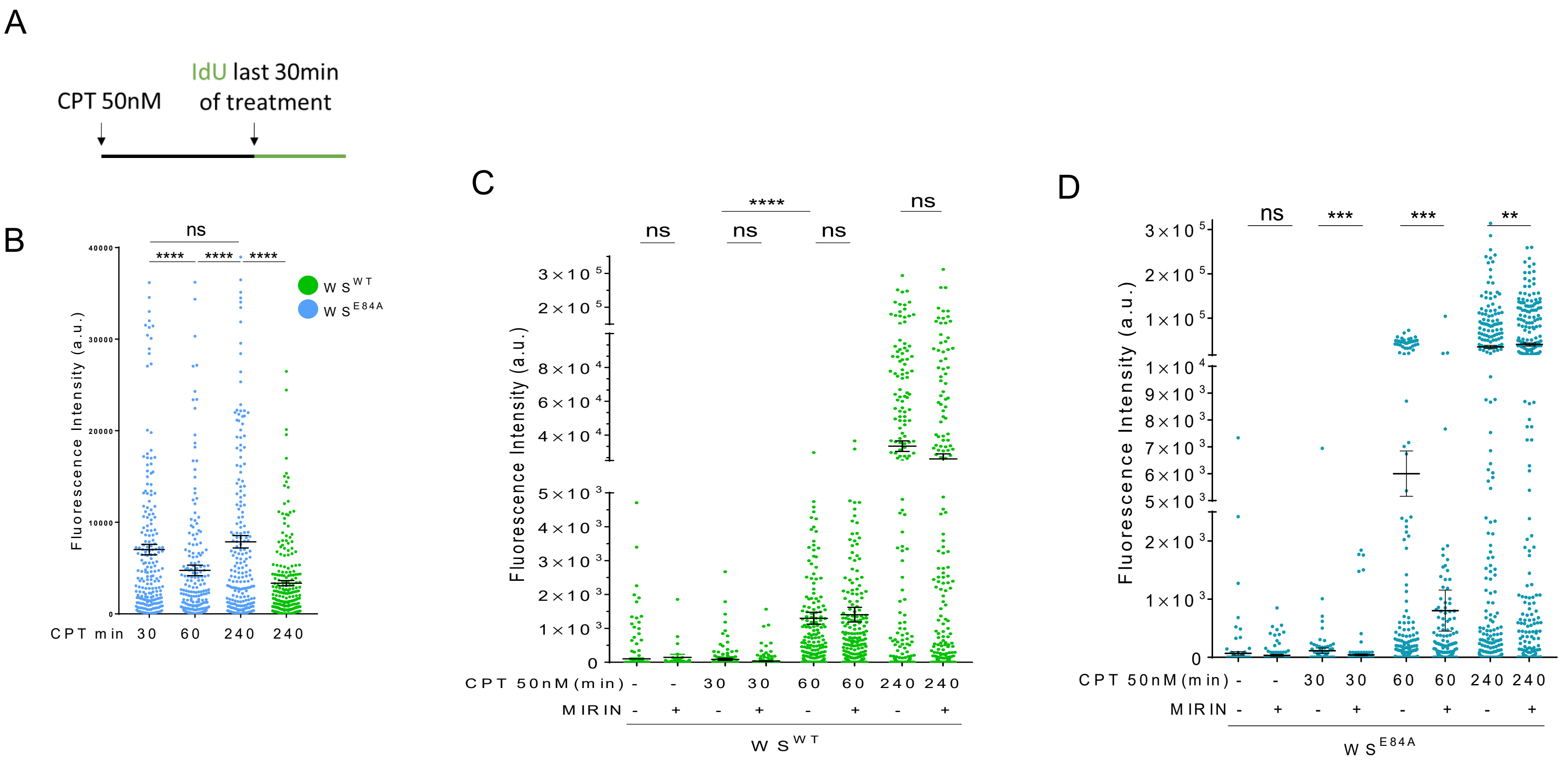

Figure S4. **MRE11 inhibition in the exo-dead mutant of WRN reduced the amount of nascent ssDNA at 30 and 60 min but not at 4h of CPT** (**A**) Experimental scheme for detection of ssDNA at nascent strands by our previous ssDNA assay. Template DNA is depicted in black, while nascent DNA is green. (**B**) Cells were either left untreated or challenged with 50 nM CPT for increasing periods, as indicated. (**C-D**) Evaluation of ssDNA by anti-IdU immunofluorescence under non-denaturing condition. Cells were treated with MIRIN 15 min before IdU labelling and 30 min before CPT treatment for 4 h and labelling remained during treatment with CPT. Dot plots show the mean intensity of ssDNA staining for single nuclei from cells expressing (**C**) the wild-type (WS^{WT}) or (**D**) the exonuclease dead form of WRN (WS^{E84A}). Cells were either left untreated or challenged with 50 nM CPT for increasing periods, as indicated. The intensity of the anti-IdU immunofluorescence was measured in at least 100 nuclei from two independent experiments. Values are represented means ±SE. Data are presented as mean ±SE. Statistical analysis was performed by the analysis of variance test (ns = not significant; $**P < 0.01$; $***P < 0.001$ $****P < 0.0001$; Mann–Whitney test)

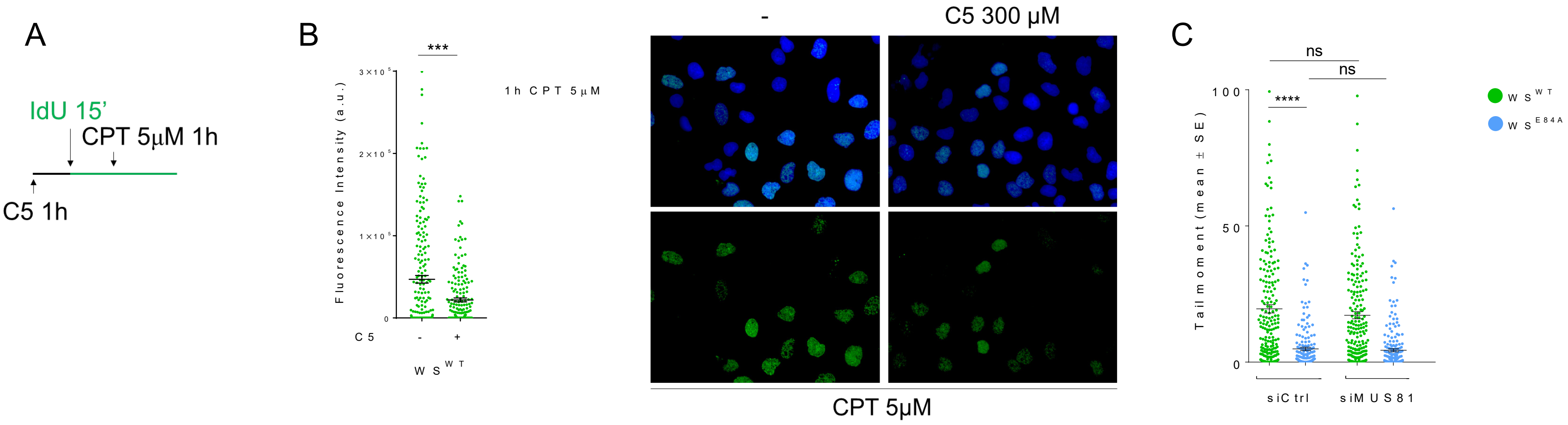

Figure S5. Functional validation of the DNA2 inhibitor C5. To confirm the ability of C5 to inhibit DNA2 in our model, we treated wild-type cells with a high-dose of CPT so that DSBs were induced. (**A**) End-resection was measured by the IdU/ssDNA assay in the presence or not of a pre-treatment with 300μM C5. The graph shows the mean intensity of IdU fluorescence measured from two independent experiments (n=200), data are presented as mean ±SE. Statistical analysis was performed by the Mann–Whitney test (****P* < 0.001). Representative images are shown in (**B**). (**C**) Analysis of DSB accumulation by the neutral Comet assay upon depletion of MUS81. Cells were transfected or not with siRNA against MUS81 for 48h and then treated with 50nM CPT for 4 h. In the graph, data are presented as mean tail moment ± SE from two independent experiments (ns = not significant; *P* < 0.0001; ANOVA test).

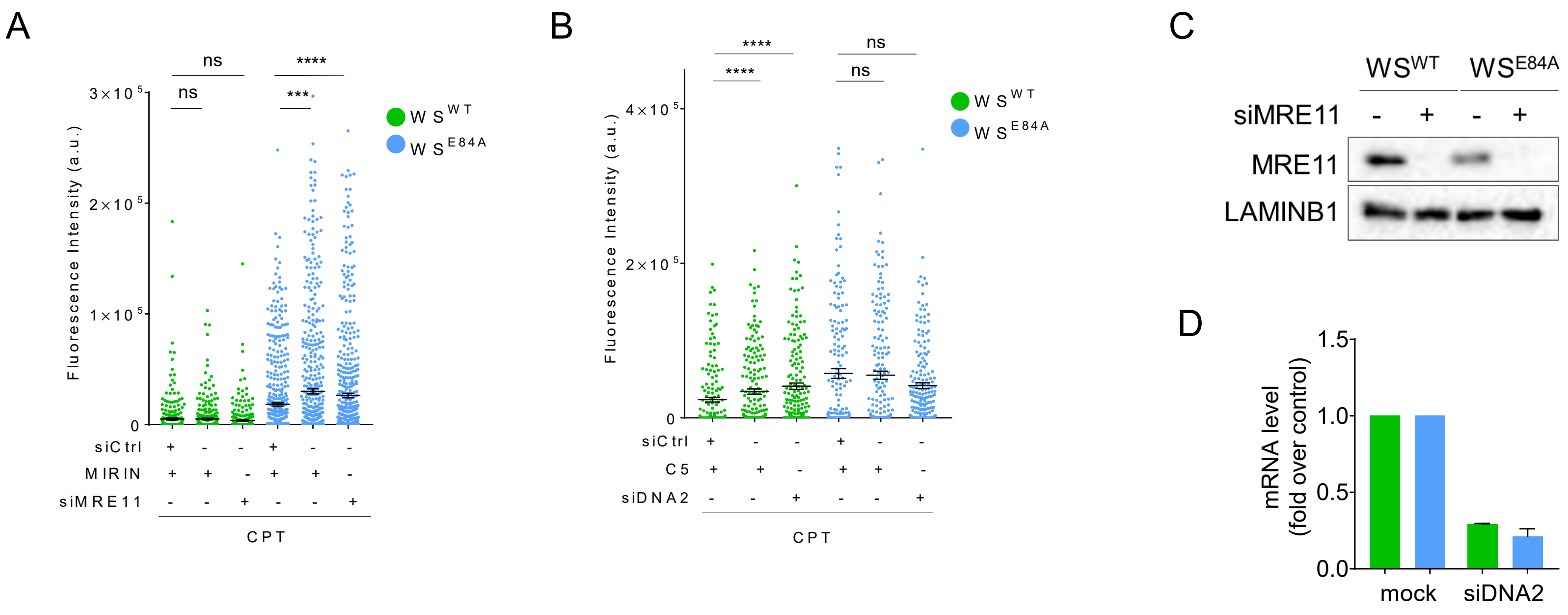

Figure S6. **Loss of exonuclease activity of WRN affects MRE11 and DNA2-dependent nascent ssDNA after CPT treatment.** Cells expressing the wild-type form of WRN(WS^{WT}) or the exo-dead form of WRN (WS^{E84A}) were transfected with Ctrl siRNAs or siRNAs against MRE11 (**A**) or DNA2 (**B**) 48 hrs prior to exposure to 50 nM CPT for 4 h (**A-B**) 48 h after transfection cells were treated or not with MIRIN (**A**) or C5 (**B**), 30 min before IdU labelling and 45 min before CPT treatment for 4 hrs, and then subjected to the ssDNA assay. Graphs show the mean intensity of ssDNA staining for single nuclei from each cell line after treatment with 50 nM CPT for 4 h. The intensity of the anti-IdU immunofluorescence was measured in at least 100 nuclei from two independent experiments. Values are represented as means ±SE. Statistical analysis was performed by the analysis of variance test (ns = not significant; ***$P < 0.001$ ****$P < 0.0001$; Mann–Whitney test). (**C**) WB analysis of MRE11 depletion was performed 48 h after transfection. (**D**) qRT–PCR analysis of DNA2 mRNA level 48 h after siCtrl or siEXO1 transfection. Expression levels were normalized *versus* two housekeeping genes, Beta-Actin and GAPDH. Relative expression levels are shown with respect to the basal level of DNA2 in cells transfected with control siRNAs, which is set equal to 1 (mean ±S.D. for $n \geqslant 2$).

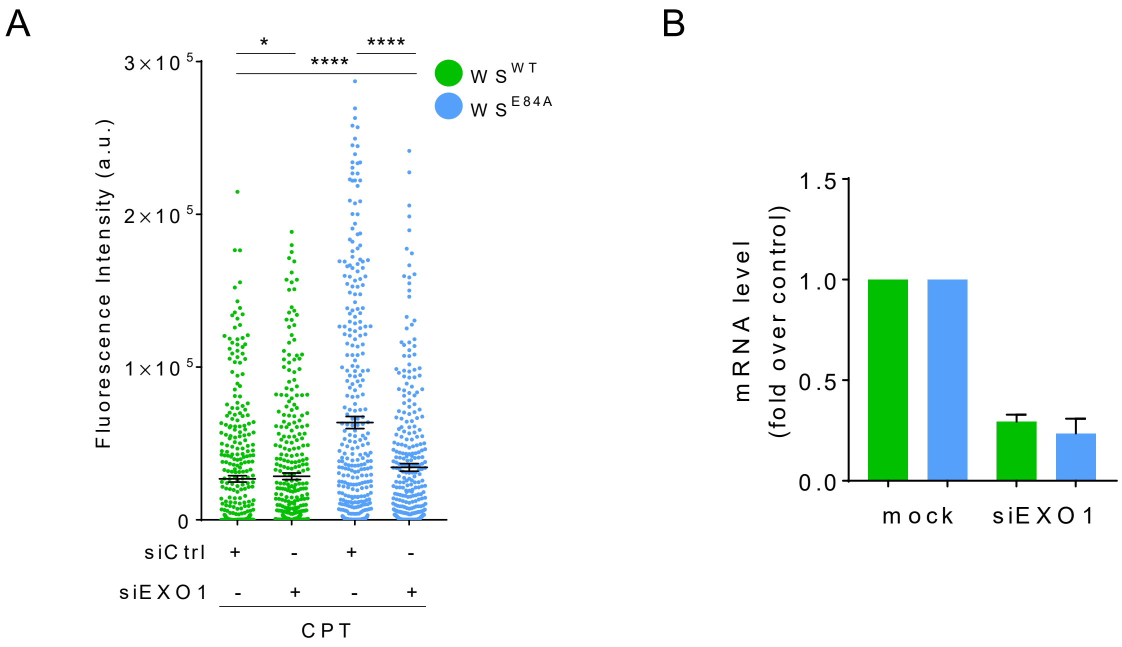

Figure S7. EXO1 depletion affects nascent ssDNA formation in WS^{E84A} cells. Cells expressing the wild-type form of WRN(WS^{WT}) or the exo-dead form of WRN (WS^{E84A}) were transfected with Ctrl siRNAs or siRNAs against EXO1 prior to exposure to 50 nM CPT for 4 h. IdU was added to cells 15 min before treatment to label nascent DNA. (**A**) Graphs show the mean intensity of ssDNA staining for single nuclei from each cell line after treatment with 50 nMCPT for 4 h. Cells were treated with the indicated RNAi at 48 hrs before the ssDNA assay. The intensity of the anti-IdU immunofluorescence was measured in at least 100 nuclei from two independent experiments. Values are represented as means ±SE. Statistical analysis was performed by the analysis of variance test (*$P < 0.05$ ****$P < 0.0001$; Mann–Whitney test). (**B**) qRT–PCR analysis of EXO1 mRNA level 48 h after siCtrl or siEXO1 transfection. Expression levels were normalized *versus* two housekeeping genes, Beta-Actin and GAPDH. Relative expression levels are shown with respect to the basal level of EXO1 in cells transfected with control siRNAs, which is set equal to 1 (mean ±S.D. for $n \geqslant 2$).

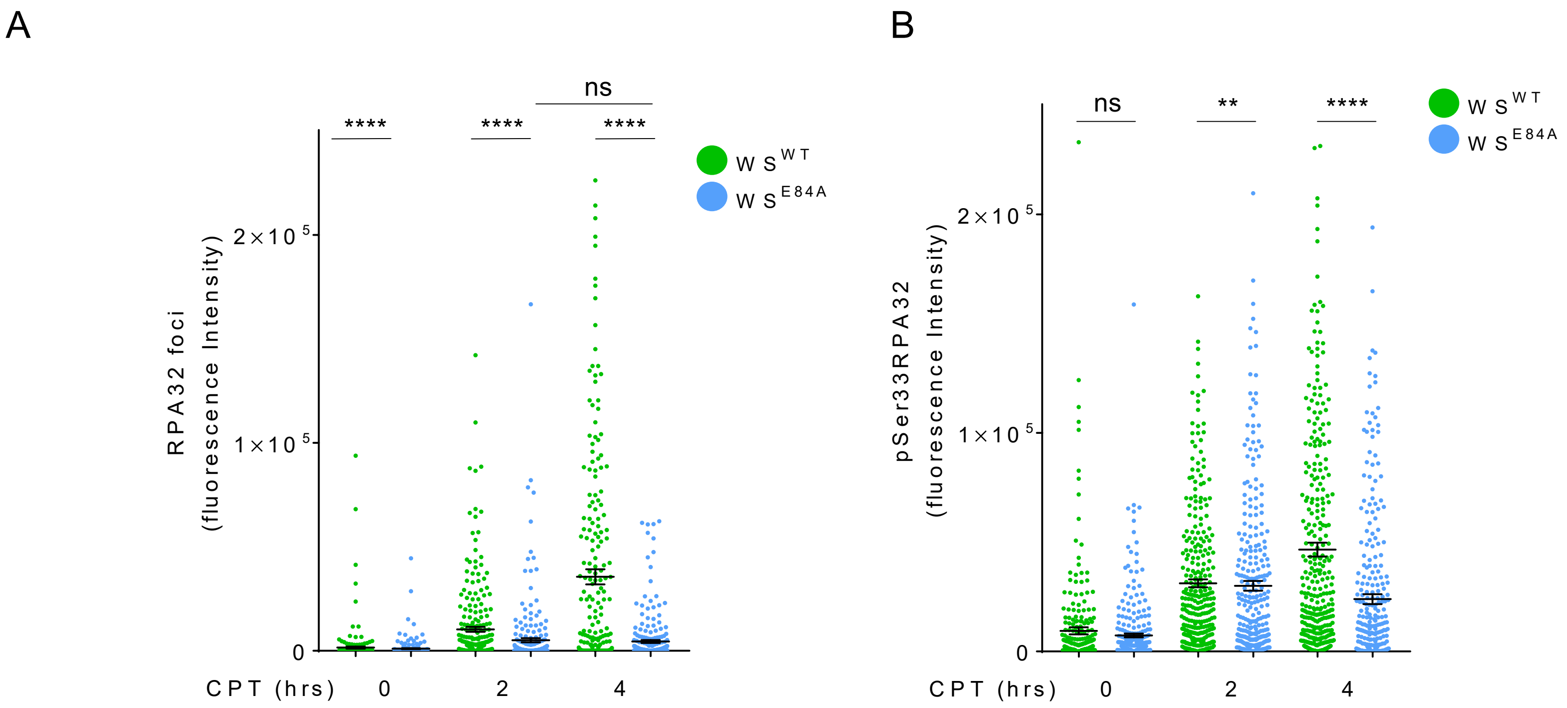

Figure S8. Analysis of ssDNA through the accumulation of RPA32 or p-S33-RPA32 foci in chromatin. Cells were treated as indicated, extracted with CSK buffer to remove non-chromatin-bound factors or loosely-bound factors, and subjected to RPA32 immunostaining. Graph shows the main intensity of RPA staining for single nuclei from untreated or treated cells. RPA intensity was measured in at least 250 nuclei from two independent experiments (A = RPA32 total; B = pS33-RPA32). Values are presented as means ± SE (ns = not significant; ** $P < 0.01$; **** $P < 0.0001$; Mann–Whitney test for $n \geqslant 2$).

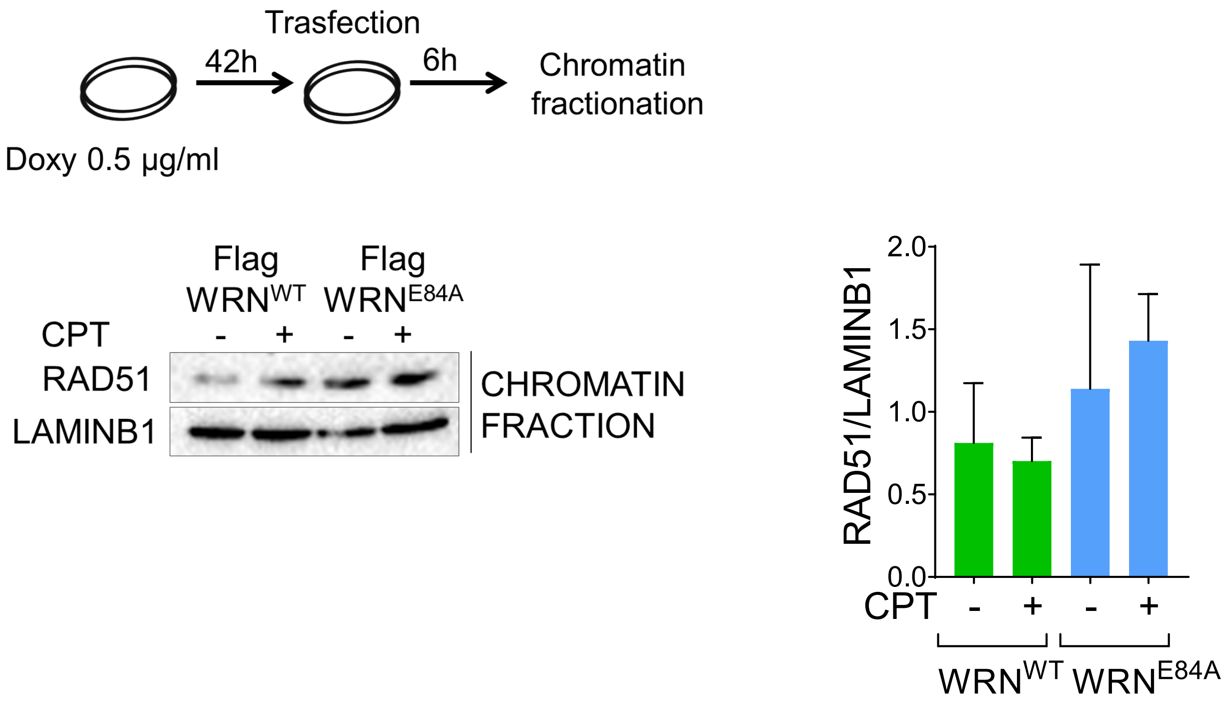

Figure S9. RAD51 recruitment to chromatin is cell line independent. Cells were treated as described in the scheme. Cells were treated with doxycycline for 48 h then was collected and used to perform chromatin fractionation. Immunoblotting shows level of RAD51 recruitment onto chromatin fraction in WS^{WT} and WS^{E84A} in untreated and treated cells. LAMIN B1 was used as loading. Graph shows the amount of RAD51 recruited in chromatin fraction.

A

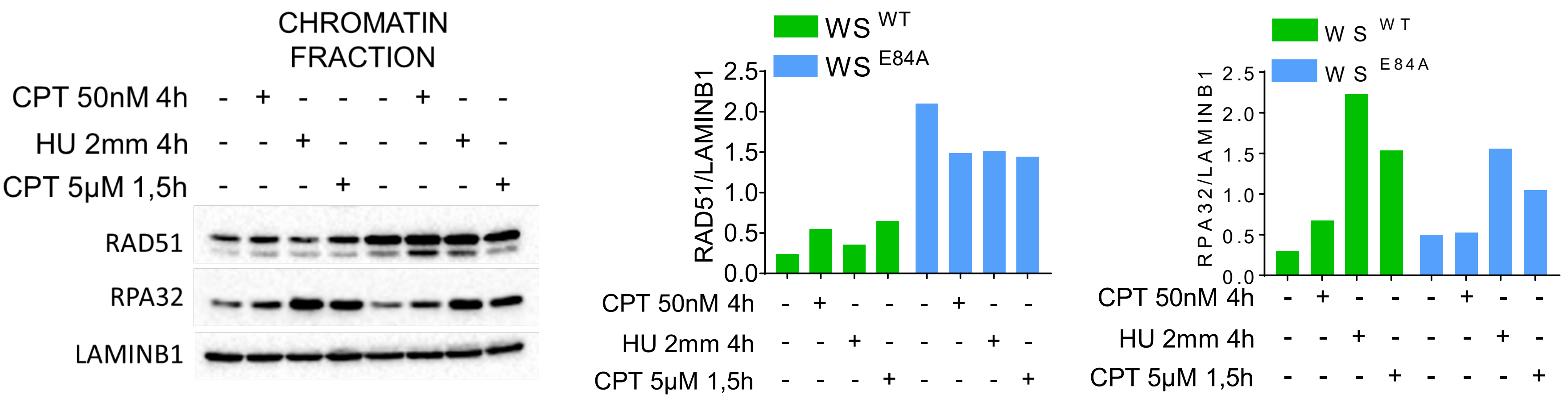

B

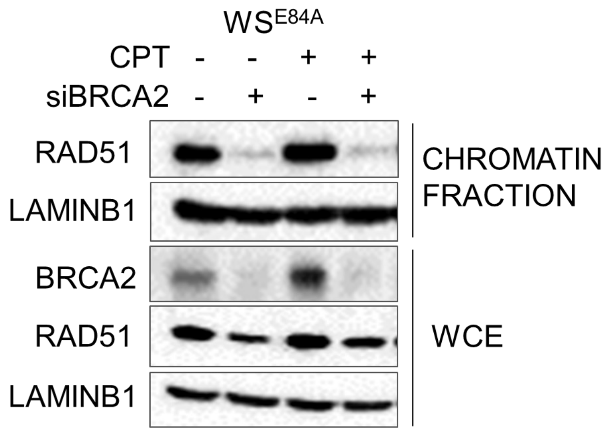

Figure S10. Analysis of RAD51 and RPA32 recruitment in chromatin after replication stress. (**A**) Evaluation of RAD51 recruitment in chromatin fraction in WS^{E84A} in untreated and treated cells. LAMINB1 was used as a loading control. Graphs show the level of RAD51 and RPA32 recruited in WS^{WT} and WS^{E84A} after different replication stress . (**B**) Cells were transfected or not with siRNA directed against BRCA2. After 48 h cells were treated or not prior to perform chromatin fractionation. Before cellular fractionation, an aliquot (10%) of cell suspension was used to reveal the amount of BRCA2 and RAD51 in all samples (WCE). LAMINB1 was used as a loading control.

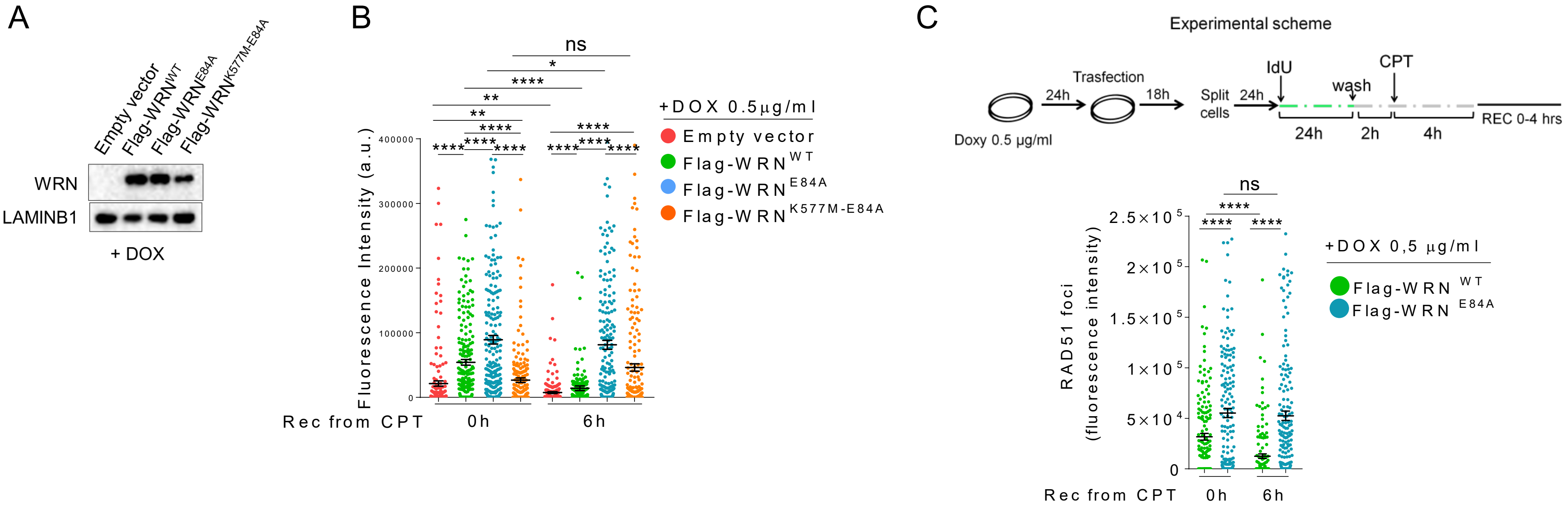

Figure S11. Accumulation of RAD51 and parental ssDNA are cell line independent and depends only on loss of the exonuclease activity of WRN (**A**) Western blot analysis shows levels of WRN after nucleofecion in doxycycline-inducible MRC5 shWRN. (**B**) Experimental scheme of transfection and parental ssDNA labeling. Graph shows the main intensity of ssDNA staining for single nuclei from untreated or treated cells. At least 100 nuclei were analyzed from two independent experiments. Values are presented as means ± SE (ns = not significant; * $P < 0.05$; ** $P < 0.01$;**** $P < 0.0001$; Mann–Whitney test). test). (**C**) Cells were transfected 48 h before RAD51 immunofluorescence. Dot plots show the mean intensity of RAD51 staining for single nuclei from each cell line after treatments. Values are presented as means ± SE (ns = not significant; **** $P < 0.0001$; Mann–Whitney test)

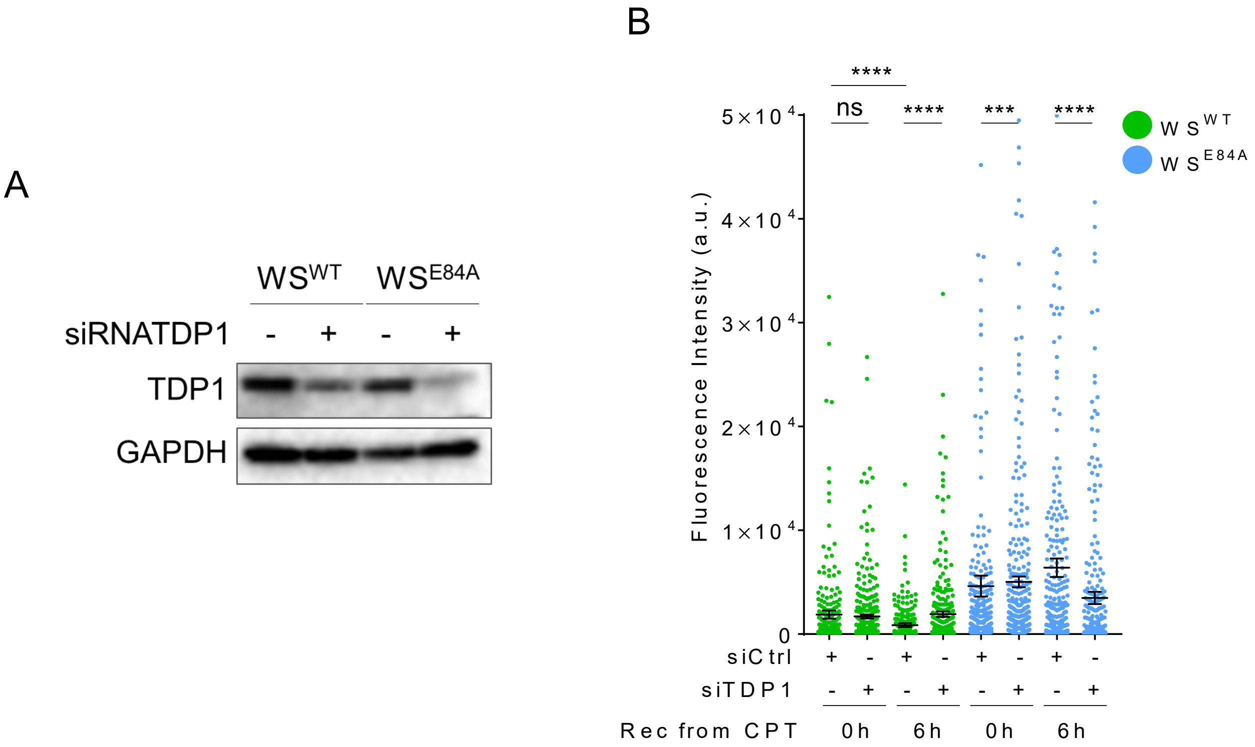

Figure S12. Analysis of parental ssDNA accumulation in cells depleted of TDP1

(A) Cells were transfected with siRNA directed against TDP1. Western blotting analysis shows level of protein. GAPDH was used as a loading control. (B) After 48 h of transfection cells were treated and ssDNA immunostaining was performed. Graph shows the main intensity of ssDNA staining for single nuclei from untreated or treated cells. Values are presented as means ± SE (ns = not significant; *** $P < 0.001$; **** $P < 0.0001$; Mann–Whitney test)

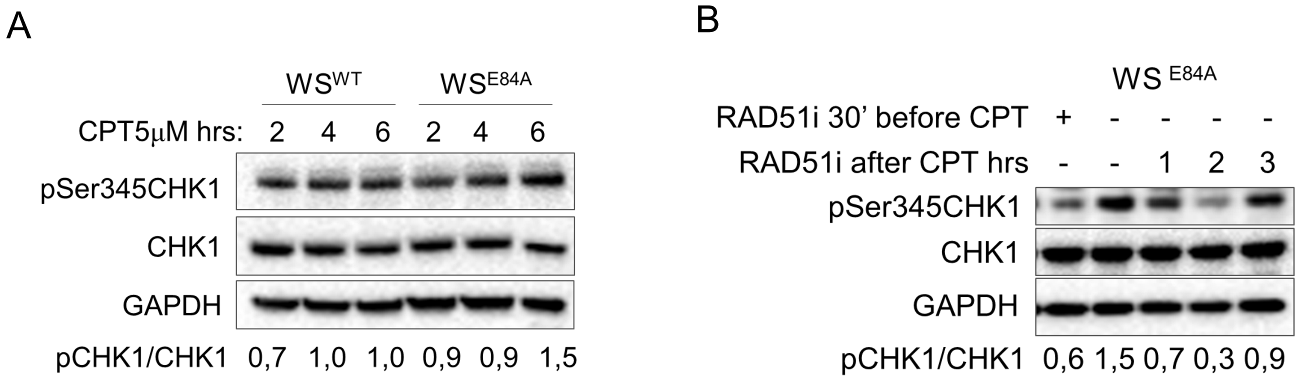

Figure S13. Evaluation of pS345CHK1 phosphorylation at high-dose of CPT and after RAD51 inhibition. (**A**) WB analysis of CHK1 phosphorylation at S345 in wild-type (WS^{WT}) and in cells expressing the exo-dead mutant form of WRN (WS^{E84A}). Cells were treated or not with a high-dose of CPT as indicated. Total CHK1 and GAPDH were used as loading control. Below are presented the quantification of CHK1 phosphorylation normalized against total CHK1. The blot is representative of two replicates. (**B**) WB analysis of CHK1 phosphorylation at S345 in cells expressing the exo-dead WRN (WS^{E84A}) upon inhibition of RAD51. Cells were treated with 50nM CPT for 4 hrs. Where indicated, RAD51i was given before CPT or in the last 3, 2 or 1hr before sampling. Total CHK1 and GAPDH were used as loading control. Below are presented the quantification of CHK1 phosphorylation normalized against total CHK1. The blot is representative of two replicates.

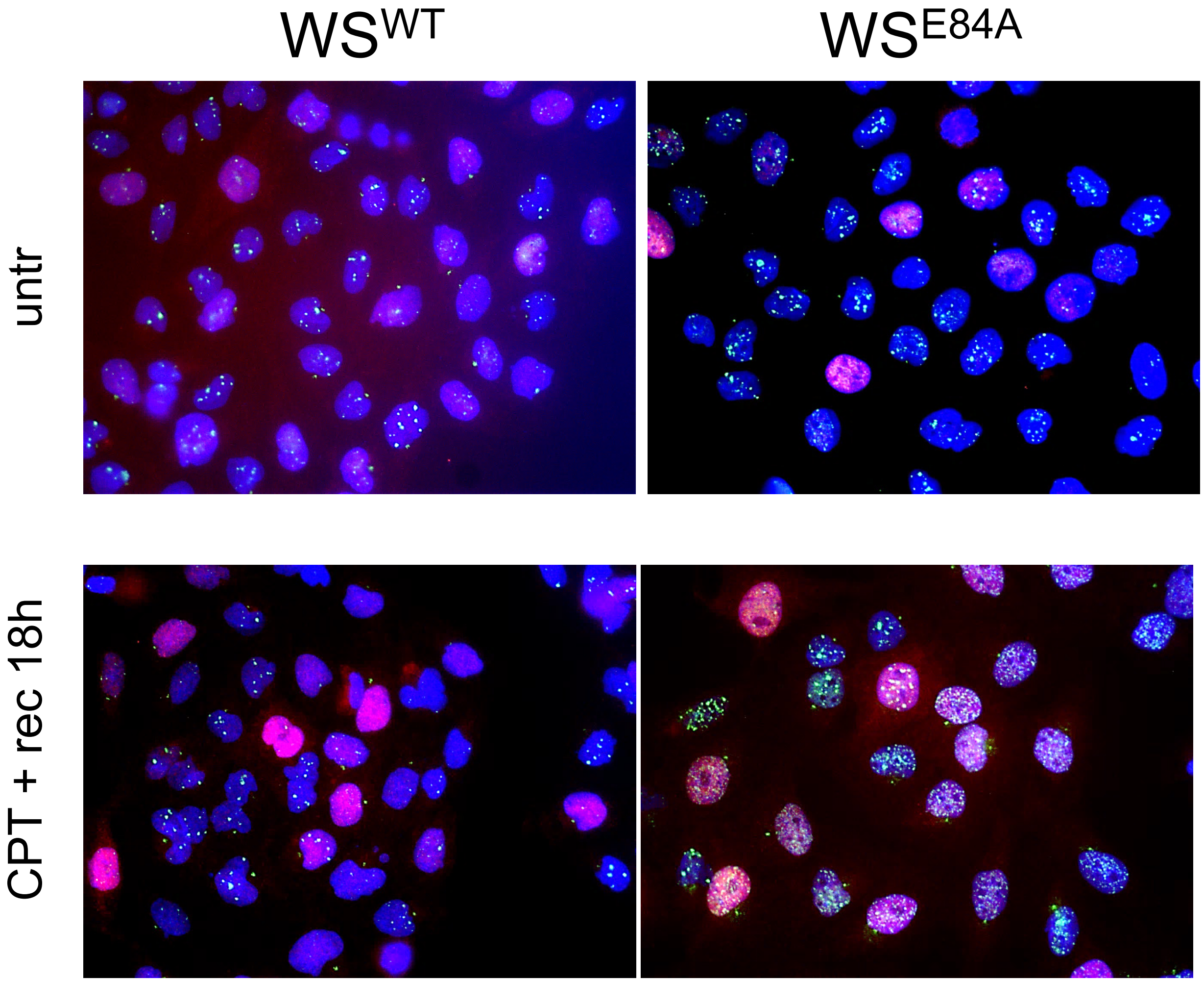

Figure S14. Immunofluorescence analysis of 53BP1 NBs. Images represent enlarged typical fields from Figure 7E.

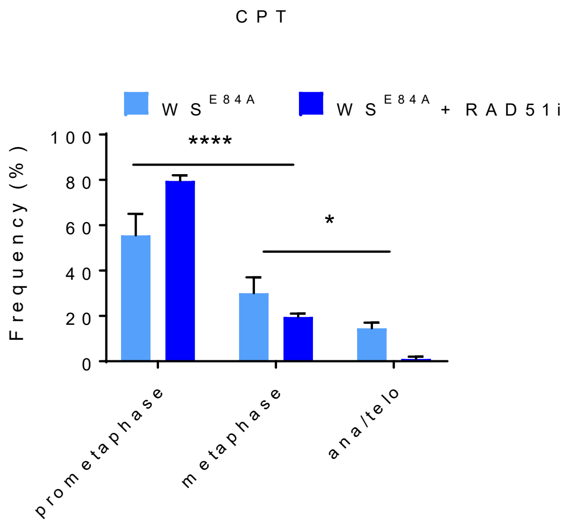

Figure S15. RAD51-inhibition induces arrest in prometaphase in WRN exonuclease-deficient cells. Cells were treated with 50nM CPT for 4h and recovered 18h in the presence or not of the RAD51i B02 prior to be analysed for the mitotic stage with anti-pS10H3 immunostaining. The graph shows the frequency of each mitotic stage presented as mean ± SE. Statistical analysis was performed by the ANOVA test (* $P < 0.05$; **** $P < 0.0001$).

SUPPLEMENTARY MATERIALS AND METHODS

Cell lines and culture conditions

The MRC5SV40 cells were maintained in Dulbecco's tet-free modified Eagle's medium (DMEM; Life Technologies) supplemented with 10% FBS tet-free (Boehringer Mannheim) and incubated at 37 °C in a humidified 5% CO_2 atmosphere. To generate the inducible shWRN cells, MRC5SV40 were transduced with lentivirus expressing an shWRN cassette under the control of a Dox-regulated promoter at 0.5 of MOI (Dharmacon SmartVector inducible lentivirus, sequence code GGAGATCAGTGGAAACTTC). After puromycin selection at 500ng/ml, a single clone was selected, tested for WRN depletion induced by doxycycline 0,5µg/ml and used throughout the study. Cell line were routinely tested for mycoplasma contamination and maintained in cultures for no more than one month.

Oligos and plasmids

pCMV-tag2B (pFLAG) empty vector was used to produce pFLAG-WRN^{WT}; pFLAG-WRN^{E84A}; pFLAG-WRN^{EK} in our laboratory.

Tranfections

MUS81 siRNA (quiagen), EXO1 siRNA and DNA2 siRNA (sigma-aldrich) were transfected at final concentration of 10nM, 25nM and 50nM respectively, using Lullaby (OZ Biosciences) 48hrs before to perform experiments.

MRE11 stealth RNAi (ACAUGUUGGUUUGCUGCGUAUUAAA; Invitrogen, Carlsbad, CA, USA) was a kind gift from Massimo Giannini (Dept. of Molecular Medicine - Sapienza University of Rome), interference was performed using a final concentration of 50nM and Lullaby as transfection reagent 60 hrs before to perform experiments.

The pCMV-tag2B (pFLAG) empty vector, pFLAG-WRN^{WT}; pFLAG-WRN^{E84A}; or pFLAG-$WRN^{E84A/K577M\ (Rnai\text{-}resistent)}$ was transfected in MRC5 shWRN cell lines using Neon transfection system 48hrs prior to perform experiments.

Chemicals

Camptothecin (Sigma-Aldrich) was dissolved in DMSO and a stock solution (10 mM) was prepared and stored at −20◦C. The final concentration for low-dose of treatments was 50nM instead 5µM of CPT was added to culture medium to induce DNA-double strand breaks. Hydroxyurea (HU) was

added to culture medium at 2 mM from stock solutions 200 mM prepared in PBS to induce DNA replication arrest or DNA damage. The B02 compound (Selleck), an inhibitor of RAD51 activity, was used at 27 µM. Clorodeoxyuridine (CldU) (Sigma-Aldrich) was dissolved in sterile water as a 200 mM stock solution and used at 50 µM. Iododeoxyuridine (IdU) (Sigma-Aldrich) was dissolved in sterile DMEM as a stock solution 2.5 mM and stored at -20 °. Mirin, the inhibitor of MRE11 exonuclease activity (Calbiochem) was used at 50 µM. DNA2-IN-C5 (MedKoo Cat#: 561402) and used at 300 µM.

Neutral Comet assay

DNA breakage induction was evaluated by Comet assay (single cell gel electrophoresis) in non-denaturing conditions. Briefly, dust-free frosted-end microscope slides were kept in methanol overnight to remove fatty residues. Slides were then dipped into molten Low Melting Point (LMP) agarose at 0.5% and left to dry. Cell pellets were resuspended in PBS and kept on ice to inhibit DNA repair. Cell suspensions were rapidly mixed with LMP agarose at 0.5% kept at 37°C and an aliquot was pipetted onto agarose-covered surface of the slide. Agarose embedded cells were lysed by submerging slides in lysis solution (30mM EDTA, 0,1% SDS) and incubated at 4°C, 1h in the dark. After lysis, slides were washed in TBE 1X running buffer (Tris 90 mM; boric acid 90 mM; EDTA 4 mM) for 1 min. Electrophoresis was performed for 20 min in TBE 1X buffer at 1 V/cm. Slides were subsequently washed in distilled H_2O and finally dehydrated in ice cold methanol. Nuclei were stained with GelRed (1:1000) and visualized with a fluorescence microscope (Zeiss), using a 60X objective, connected to a CCD camera for image acquisition. At least 300 comets per cell line were analyzed using CometAssay IV software (Perceptive instruments) and data from tail moments processed using Prism software. Apoptotic cells (smaller Comet head and extremely larger Comet tail) were excluded from the analysis to avoid artificial enhancement of the tail moment.

In situ PLA assay for ssDNA-protein interaction

The *in situ* proximity ligation assay (PLA; Olink, Bioscience) was performed according to the manufacturer's instructions. For nascent ssDNA-protein interaction cells were labeled with 100µM IdU for 15 minutes before treatments. For parental ssDNA-protein interaction cells were labeled with 100µM IdU for 20 h and released in fresh medium for 2h before treatments. After treatment, cells were permeabilized with 0.5% Triton X-100 for 10 min at 4°C, fixed with 3% formaldehyde/2% sucrose solution for 10 min and then blocked in 3% BSA/PBS for 15min. After

washing with PBS, cells were incubated with the two relevant primary antibodies. The primary antibodies used were as follows: rabbit polyclonal anti-RAD51 (Abcam, 1:500, anti-IdU (mouse monoclonal anti-BrdU/IdU; clone b44 Becton Dickinson, 1:40). The negative control consisted of using only one primary antibody. Samples were incubated with secondary antibodies conjugated with PLA probes MINUS and PLUS: the PLA probe anti-mouse PLUS and anti-rabbit MINUS (OLINK Bioscience). The incubation with all antibodies was accomplished in a humidified chamber for 1 h at 37°C. Next, the PLA probes MINUS and PLUS were ligated using two connecting oligonucleotides to produce a template for rolling-cycle amplification. After amplification, the products were hybridized with red fluorescence-labelled oligonucleotide. Samples were mounted in Prolong Gold anti-fade reagent with DAPI (blue). Images were acquired randomly using Eclipse 80i Nikon Fluorescence Microscope, equipped with a Video Confocal (ViCo) system.

EdU incorporation assay

To label replicated DNA, cells were incubated with 10 µM EdU for 30 minutes. Samples were fixed with 4% PFA at RT for 10 min and cells were subsequently permeabilized with 0,5% Triton-X100. EdU incorporation was detected using the Click-It Edu Alexa Fluor 488 Imaging Kit (Invitrogen) according to the manufacturer’s instructions.

In situ PLA assay for detection of protein at replication fork (SIRF)

Exponential growing cells were seeded onto microscope chamber slide. The day of experiment, cells were incubated with 125 µM EdU for 8 min, then were washed with PBS for 5 min and treated as indicated. After treatments cells were fixed with 2%PFA/PBS, washed in PBS two times for 5 min each and permeabilized with 0.25% TritonX-100 in PBS for 15 min at room temperature. Clik it reaction cocktail was freshly preparated and slides were incubated at room temperature for 30 min. After clik it reaction, slides were washed in PBS for 5 min and placed back in the humid chamber with blocking solution (3% BSA/PBS) for 15 min. The primary antibodies used were as follows: rabbit monoclonal anti-WRN (Abcam 1:300), and Biotin (Invitrogen, 1:500). The negative control consisted of using only one primary antibody. Samples were incubated with secondary antibodies conjugated with PLA probes MINUS and PLUS: the PLA probe anti-mouse PLUS and anti-rabbit MINUS (OLINK Bioscience). The incubation with all antibodies was accomplished in a humidified chamber for 1 h at 37°C. Next, the PLA probes MINUS and PLUS were ligated using two connecting oligonucleotides to produce a template for rolling-cycle amplification. After amplification, the products were hybridized with red fluorescence-labelled oligonucleotide.

Samples were mounted in Prolong Gold anti-fade reagent with DAPI (blue). Images were acquired randomly using Eclipse 80i Nikon Fluorescence Microscope, equipped with a Video Confocal (ViCo) system.

gene expression analysis

Relative mRNA was analyzed by absolute QPCR using TaqMan probes. Nuclear DNA was detected by using EXO1 and DNA2 single tube Taqman real-time PCR assay (cat. no. 44492; Life Technologies). RNA was extracted by RNeasy kit (cat. no. R1054; Zymo Research) and cDNA synthesis was carried out by the high capacity cDNA reverse transcription kit (cat. no. 4368813; Life Technologies). Gene expression analysis was carried out using single tube Taqman real-time PCR assays (cat. no. 4331182; Life Technologies).
